# Correction: The Multitentaculate Cirratulidae of the Genera *Cirriformia* and *Timarete* (Annelida: Polychaeta) from Shallow Waters of Brazil

**DOI:** 10.1371/journal.pone.0116130

**Published:** 2014-12-19

**Authors:** The PLOS ONE Staff

## Notice of Republication

This article was republished on November 21st, 2014, due to multiple figures being posted incorrectly. The publisher apologizes for this error. Please download this article again to view the corrected version. The originally published, uncorrected article and the republished, corrected article are provided here for reference.

## Supporting Information

File S1 Originally published, uncorrected article
(PDF)

File S2 Republished, corrected article
(PDF)
