# Supplementary material for: Correction: The Multitentaculate Cirratulidae of the Genera Cirriformia and Timarete (Annelida: Polychaeta) from Shallow Waters of Brazil
Source: PLoS One. 2014 Dec 19;9(12):e116130. doi: 10.1371/journal.pone.0116130 (PMC4272290; doi:10.1371/journal.pone.0116130)
Supplement: File S1 — Originally published, uncorrected article (PDF) [file pone.0116130.s001.pdf]

The Multitentaculate Cirratulidae of the Genera *Cirriformia* and *Timarete* (Annelida: Polychaeta) from Shallow Waters of Brazil

Wagner F. Magalhães[1]*, Victor Corrêa Seixas[2], Paulo Cesar Paiva[2], Rodolfo Elias[3]

1 Department of Biology, University of Hawaii at Manoa, Honolulu, Hawaii, United States of America, **2** Departamento de Zoologia, Universidade Federal do Rio de Janeiro, Rio de Janeiro, Rio de Janeiro, Brazil, **3** Departamento de Ciencias Marinas, Universidad Nacional de Mar del Plata, Mar del Plata, Buenos Aires, Argentina

Abstract

A large number multitentaculate cirratulids have been described worldwide but most are only known through the original descriptions. Type material, voucher and recently collected specimens from Brazil were revisited in order to reveal their true identity and confirm the records of widely distributed species in this region. Six species are described, three of which are new, *Cirriformia capixabensis* sp. nov., *Cirriformia chicoi* sp. nov. and *Timarete ceciliae* sp. nov. COI and 16S sequences were obtained and used for inter-specific comparisons. *Timarete caribous* is reported from several localities along the Brazilian coast and a new synonym, *Cirratulus melanacanthus,* is proposed. The species *Timarete oculata*, originally described from Brazil and lumped into the *Timarete filigera* species complex, is herein revalidated and redescribed. The occurrence of the species *Timarete filigera* and *Cirriformia tentaculata* is not confirmed from the Brazilian coast. Descriptions, illustrations and a key to genera and species are provided.

Citation: Magalhães WF, Seixas VC, Paiva PC, Elias R (2014) The Multitentaculate Cirratulidae of the Genera *Cirriformia* and *Timarete* (Annelida: Polychaeta) from Shallow Waters of Brazil. PLoS ONE 9(11): e112727. doi:10.1371/journal.pone.0112727

Editor: Helge T. Lumbsch, Field Museum of Natural History, United States of America

Received August 6, 2014; **Accepted** October 10, 2014; **Published** November 13, 2014

Copyright: © 2014 Magalhães et al. This is an open-access article distributed under the terms of the Creative Commons Attribution License, which permits unrestricted use, distribution, and reproduction in any medium, provided the original author and source are credited.

Data Availability: The authors confirm that all data underlying the findings are fully available without restriction. All relevant data are within the paper.

Funding: The work was supported by the following: PP VS - Conselho Nacional de Desenvolvimento Científico e Tecnológico (CNPq), Coordenadoria de Aperfeiçoamento de Pessoal de Nível Superior (CAPES) and Fundação de Amparo à Pesquisa do Estado do Rio de Janeiro (FAPERJ). The funders had no role in study design, data collection and analysis, decision to publish, or preparation of the manuscript.

Competing Interests: The authors have declared that no competing interests exist.

* Email: wagnerfm@hawaii.edu

Introduction

The multitentaculate cirratulids form a clade composed of five genera, *Cirratulus* Lamarck, 1801, *Timarete* Kinberg, 1866, *Protocirrineris* Czerniavsky, 1881, *Cirriformia* Hartman, 1936, and *Fauvelicirratulus* Çinar & Petersen, 2011. All of these genera possess more than a single pair of tentacles attached on the dorsal part of anterior segments. These dorsal tentacles are either organized in longitudinal rows as in *Protocirrineris* species or in transverse or oblique groups in the remaining genera. The number, segmental origin and distribution of branchial filaments are also of generic interest. *Timarete* species usually have branchiae originating from the posterior end of the peristomium, anterior to the dorsal tentacles and these branchiae shift to a more dorsal location, posteriorly. The branchial filaments in *Cirriformia* species remain near the notopodial lobes throughout the body. Branchiae in species of *Cirratulus* begin on the same segment as the tentacular filaments, usually chaetiger 1. The recently described genus *Fauvelicirratulus* has a similar arrangement of branchiae and tentacles as in *Cirratulus* but differs by the presence of more than a single pair of branchiae per segment and by different types of spinous chaetae.

Several multitentaculate species have been described from South America but most have been invalidated or synonymized. *Cirratulus melanacanthus* Fr. Müller & Grube in Grube, 1872 was described from Desterro, southern Brazil and includes the species *Cirratulus danielsi* Hansen, 1882 also described from Brazil as its junior synonym [1,2,3]. *Cirratulus flavescens* Grube, 1872 and *Cirratulus obscurus* Quatrefages, 1866 were described from Southern Brazil but later considered as homonyms [1,2,4]. *Cirratulus jucundus* (Kinberg, 1866), *Cirratulus patagonicus* (Kinberg, 1866) and *Cirriformia nasuta* Ehlers, 1897 were described from Argentina and are all currently considered as valid [1,5,6]. The species *Audouinia oculata* Treadwell, 1932 was described for southern Brazil and later placed in *Timarete filigera* [1,7].

Most of the recent literature on multitentaculate cirratulids from Brazil has referenced *Timarete filigera* and *Cirriformia tentaculata* (Also referred to as *Timarete tentaculata*) [8] but these are Mediterranean and northern Atlantic species and unless it was a human-mediated introduction, it is unlikely to occur in South America. For this reason, we investigate herein the identity of multitentaculate Cirratulidae from Brazil through re-examination of types, voucher material and recently collected specimens along the Brazilian coast. This study is the first attempt to review the multitentaculate cirratulids from the Brazilian coast and serves as a baseline for future studies concerning this diverse and complex group.

Materials and Methods

Specimens from several localities along the Brazilian coast (Fig. 1) were collected in soft sediment, macroalgae and coralline

algae along the intertidal zones; sediment was collected with a shovel and sieved through a 0.5 mm screen. Specimens collected in Salvador, northern Brazil (Fig. 1) were observed and photographed live after anesthetization in 8% $MgCl_2$ for a few minutes. Most of the specimens used for morphological analyses were fixed in 5% formalin for at least 24 hours and transferred to 70% ethanol. Molecular analyses were carried out with specimens preserved in 92.8% ethanol.

Samples from São Pedro e São Paulo Archipelago were collected in the subtidal zone by SCUBA or free diving. A collecting permit for the marine protected areas (i.e. São Pedro e São Paulo Archipelago, Fernando de Noronha and Rocas Atoll) was granted by the Instituto Chico Mendes de Conservação da Biodiversidade (ICMBio) under permit number 23760-2 to P.C. Paiva. Sampling in unprotected areas (including outside the National Park of Abrolhos Atoll) were under permit number 10238-1, also granted by ICMBio to P.C. Paiva.

Type material and voucher specimens of cirratulids described or reported from South America were borrowed from the United States National Museum of Natural History, Smithsonian Institution, Washington, D. C., USA (USNM) and Museum für Naturkunde, Berlin, Germany (MfN). Holotypes and paratypes are deposited at the Polychaete Collection "Edmundo Ferraz Nonato" – Instituto de Biologia, Universidade Federal do Rio de Janeiro (IBUFRJ).

Specimens were examined using a phase contrast light microscope and some with a Scanning Electron Microscope (SEM). Line drawings were made with a camera lucida attached to a compound microscope and length and width measurements were taken with an ocular micrometer that was calibrated with a stage micrometer. SEM preparations included dehydration through a series of increasing concentrations of ethanol ending with two changes of absolute ethanol followed by critical point drying (in a SAMDRI-795). Worms were mounted on stubs and coated with gold/palladium for two minutes at 5 nm thickness. SEM observations were carried out using the Hitachi S-4800 at the Biological Electron Microscopy Facility (BEMF), University of Hawaii at Manoa.

Genomic DNA was extracted with a Puregene Core Kit A (Gentra Systems) or according to the protocol developed for nematodes by [9]. Fragment sequences of two mitochondrial markers were obtained via polymerase chain reaction (PCR). Cytochrome oxidase subunit I (COI) was amplified using universal primers, LCO1490 and HCO2198 [10]. Each 25 μL reaction included 1X *Taq* buffer, 3.0 mM of magnesium chloride, 1 mg/ml of Bovine Serum Albumin (BSA), 0.2 mM of dNTPs, 0.6 μM of each primer, 1 unit *Taq* DNA polymerase and 1 μL of extracted DNA. PCR conditions were one cycle of 94°C for 2 min, five cycles of 95°C for 40 s, 45°C for 90 s and 72°C for 1 min, followed by 35 cycles of 95°C for 40 s, 51°C for 45 s and 72°C for 1 min, and a final extension step of 72°C for 5 min. 16S ribosomal RNA (16S) was amplified using a forward primer 16Sar-L [11] and a reverse primer 16SAN-R [12]. Each 25 μL reaction included 1X *Taq* buffer, 3.0 mM of magnesium chloride, 0.4 mg/ml of BSA, 0.12 mM of dNTPs, 0.6 μM of each primer, 1 unit *Taq* DNA polymerase and 1 μL of extracted DNA. PCR conditions were one cycle of 94°C for 3 min, 35 cycles of 94°C for 30 s, 46°C for 30 s and 72°C for 90 s, and a final extension step of 72°C for 7 min. The purification and sequencing were conducted by Macrogen Inc. (Korea, Seoul) and using an AB3500 automated sequencer (Applied Biosystems) in the Laboratório de Biodiversidade Molecular, Federal University of Rio de Janeiro (Brazil).

The electropherograms were edited with Sequencher 4.1 software (Gene Codes Corporation) and sequences were aligned in MEGA 5.0 [13] with ClustalW tool. The number of divergent sites and the intra and inter-specific genetic distances were also calculated with MEGA 5.0 [13]. Sequences were deposited in the GenBank database (www.ncbi.nlm.nih.gov/genbank).

Nomenclatural Acts

The electronic edition of this article conforms to the requirements of the amended International Code of Zoological Nomenclature, and hence the new names contained herein are available under that Code from the electronic edition of this article. This published work and the nomenclatural acts it contains have been registered in ZooBank, the online registration system for the ICZN. The ZooBank LSIDs (Life Science Identifiers) can be resolved and the associated information viewed through any standard web browser by appending the LSID to the prefix "http://zoobank.org/". The LSID for this publication is: urn:lsid:zoobank.org:pub: 2469618D-EEF2-49F8-95A9-E74C0B83F65E. The electronic edition of this work was published in a journal with an ISSN, and has been archived and is available from the following digital repositories: PubMed Central, LOCKSS.

Taxonomic Account

Key to genera and species of multitentaculate Cirratulidae from South America

1 Chaetae all capillaries, first pair of branchial filaments arise on chaetigers 3 or 4; tentacular filaments arranged in two groups of 2–3 longitudinal rows each side of 4–7 segments beginning on chaetigers 4 or 5 ......*Protocirrineris antarcticus**.

– Chaetae include capillaries and acicular spines ....2.

2(1) Segmental origin of branchial filaments on same chaetiger as dorsal tentacles, usually chaetiger 1 (*Cirratulus*) ....3.

– Segmental origin of branchial filaments anterior to dorsal tentacles ....4.

3(2) Eyes present, organized in postero-lateral rows of eight dark eyespots in adults and smaller numbers in juveniles; branchial filaments from chaetiger 1 arising with dorsal to notopodial base, becoming more dorsal on posterior segments but not reaching mid-dorsum; tentacular filaments in two oblique groups arising between posterior end of peristomium and posterior end of chaetiger 1 ....*Cirratulus jucundus**.

– Eyes present, organized in postero-lateral rows of six pale eyespots; first pair of branchial filaments arising from posterior end of chaetiger 1; subsequent branchiae shifted to mid-dorsum from chaetigers 2–4; with two transverse groups of tentacular filaments arise above chaetiger 1 ....*Cirratulus patagonicus**.

4(2) Branchiae arising from near notopodial base throughout body (*Cirriformia*) ....5.

– Branchiae shifting to mid-dorsum on mid-body segments (*Timarete*) ....6.

5(4) Peristomium with three sub-equal annulations and no sub-annulations; groups of tentacles above two segments (2–3, 3–4 or 4–5); notopodial spines from chaetigers 31–42 and neuropodial spines from 19–24; methyl green staining pattern forming transversal bands of green speckles on posterior half of body segments ....*Cirriformia chicoi,* sp. nov.

– Peristomium with three sub-equal annulations, all with 3–4 sub-annulations; groups of tentacles above two segments (5–6 or 6–7); notopodial spines from chaetigers 45–55 and neuropodial spines from 34–40; methyl green staining pattern forming transversal bands of green speckles on segments leaving only inter-segmental regions unstained ....*Cirriformia capixabensis,* sp. nov.

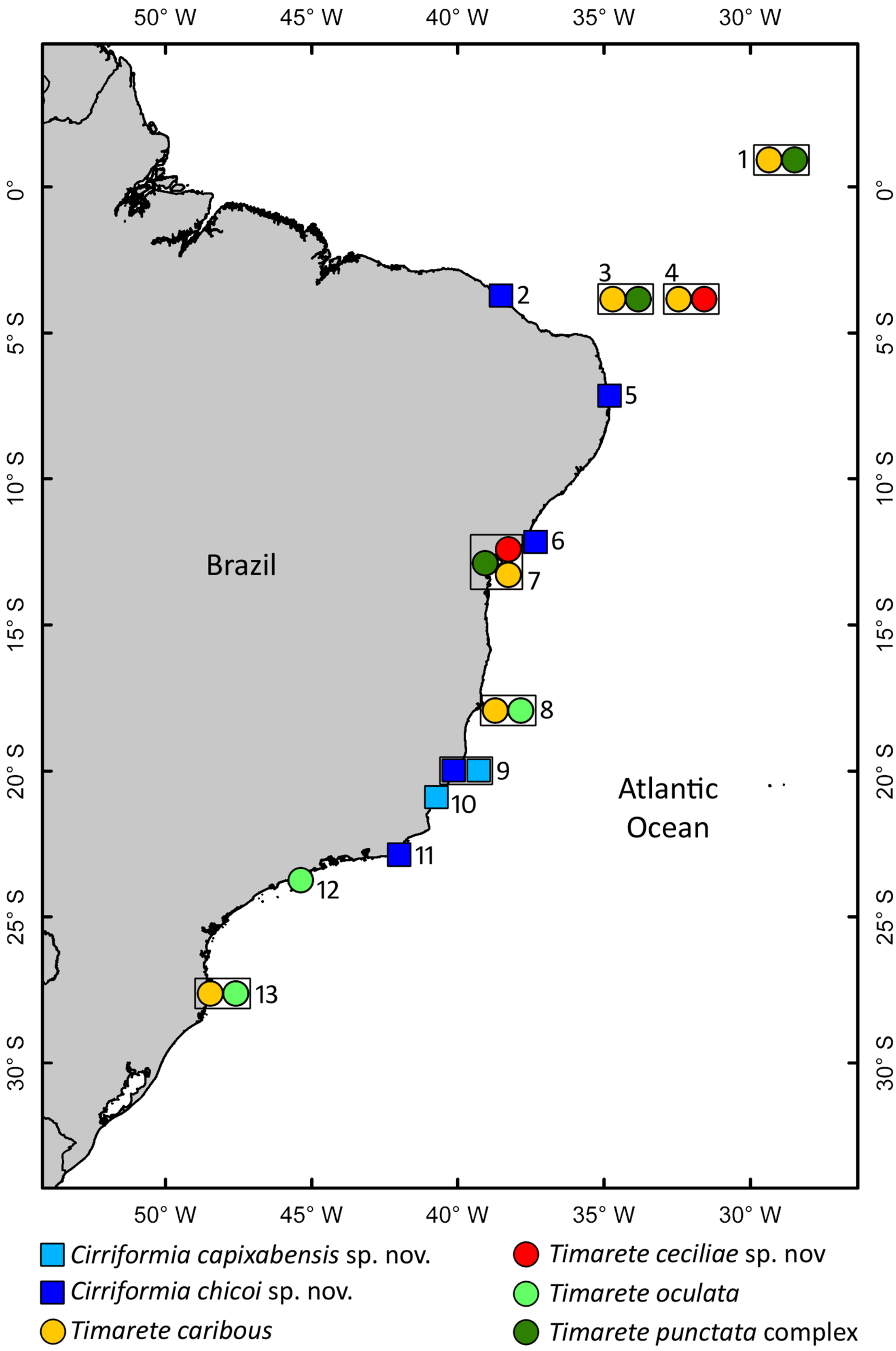

Figure 1. Map showing distribution of multitentaculate cirratulids along the Brazilian coast. Symbols within the same square indicate that the species were collected in the same locality. Localities are numbered as follow: 1. São Pedro e São Paulo Archipelago; 2. Fortaleza; 3. Rocas Atoll; 4. Fernando de Noronha Archipelago (Sueste beach); 5. João Pessoa (Cabo Branco beach); 6. Salvador (Itapuã beach); 7. Salvador (Ribeira beach); 8. Abrolhos Archipelago; 9. Vitória (Boi Island); 10. Guarapari; 11. Cabo Frio (Japonês Island); 12. São Sebastião; 13. Florianópolis.
doi:10.1371/journal.pone.0112727.g001

6(4) Preserved specimens without pigmentation on tentacles and branchiae and only lightly pigmented on ventral region of anterior end ....7.

– Preserved specimens with dark brown lateral stripes on tentacles and branchiae and dark spots throughout the body ....*Timarete punctata* species complex.

7(6) Branchial filaments on mid-body and posterior chaetigers shifted to high on body wall above notopodia; distance between same segment branchial pairs smaller than distance in between each filament and notopodial base; posterior neuropodia with single large, dark spine on posterior segments lacking companion capillaries ....*Timarete caribous*.

– Branchial filaments shifted to mid-dorsum but gap between same segment branchial pairs always larger than distance in between each filament and notopodial base; posterior neuropodia usually with 2–3 spines ....8.

8(7) Branchiae shifted gradually towards mid-dorsum from chaetigers 12–14 forming a bulge over notopodia; two transversal groups of about 15–20 tentacular filaments on chaetigers 4–5; methyl green staining pattern distinct on peristomium, dorsum of chaetigers 1–3 and parapodial region ....*Timarete nasuta**.

– Branchiae shifted gradually towards mid-dorsum but not forming bulge over notopodia ....9.

9(8) Branchiae shifted gradually towards mid-dorsum from chaetigers 20–25; groups of tentacles on chaetigers 4–5 or 6–7; methyl green staining pattern distinct on prostomium, peristomium and parapodial regions, body uniformly stained ....*Timarete oculata*.

– Branchiae shifted gradually towards mid-dorsum from chaetigers 7–14; groups of few tentacles on chaetigers 2–3, 3–4 or 4–5; methyl green staining pattern distinct with branchiae and pygidium speckled and segmental regions with stain encircling individual segments forming complete rings ....*Timarete ceciliae* sp. nov.

---

*These species are not included in this study and have not been recorded to Brazilian waters

Descriptions

Cirriformia Hartman, 1936

Type Species: Terebella tentaculata Montagu, 1804, designated by Hartman, 1936.

Diagnosis (After Blake, 1996): Prostomium elongate or blunt, usually without eyes; peristomium with 2–3 annulations. Grooved tentacular filaments limited to 1–3 anterior segments, arising between chaetigers 2–7. Branchiae occurring singly, usually first present from chaetiger 1, arising close to notopodia throughout, not shifting dorsal in middle body segments and not forming dorso-lateral branchial bulges. Parapodia rami well-separated. Chaetae including capillaries and acicular spines.

Cirriformia capixabensis, sp. nov. [urn:lsid:zoobank.org:act:5E875347-138A-458D-A6CF-123B96AA49B2]
Figures 2 and 3 (B).

Material examined

Type material: Holotype: Brazil, Espírito Santo, Guarapari, 20° 39′ 46" S, 40° 29′ 49.3" W, Dec/1/2013, coll. A.M. Lanna (IBUFRJ-3780). Paratypes: same locality, date and collector as holotype (2, IBUFRJ-3561).

Non-type material: Brazil: Espírito Santo, Vitória, Boi Island, 20° 18′ 35.7" S, 40° 17′ 3.46" W, Apr/08/2012, coll. B. Silva (1 incomplete, IBUFRJ-3562).

Comparative material: *Cirriformia tortugaensis* (Augener, 1922), Tortugas, Bird Key Riff, leg. Hartmeyer (type MfN 6405), two slides (MfN 6405a, 6405b); *Cirriformia chefooensis* (Grube, 1877), Chefoo, China, coll. Grube (6, MfN Q4527); *Cirriformia afer* (Ehlers, 1908), Grobe Fischbei, Stat. 77, leg. Valdivia, (1 anterior end, MfN 4483), Gr. Fischbei, Valdivia Exp. Ded: Zool. Inst. Göttingen, (3 anterior ends+2 posterior ends, MfN 6756).

Description

Holotype 55 mm long, 2 mm wide for 215 chaetigers. Two paratypes 50–52 mm long, 1.8–2 mm wide for 210–220 chaetigers. Body elongated, rounded dorsally, flattened ventrally; parapodia form lateral shoulders on thoracic region. Color in alcohol white to pale yellow. Pygidium with ventral lobe, anal aperture placed dorso-terminally.

Prostomium conical, as long as two anterior chaetiger, with pair of large postero-lateral nuchal organs, as deep depressions (Fig. 2A). Peristomium elongate, as long as four anterior chaetigers with three similar-sized annulations; posterior two annuli with 3–4 sub-annulations (Fig. 2A). Branchial filaments first present on posterior end of chaetiger 1, one pair per segment, present in most segments on first one-third of body, few afterwards, absent on last 20–30 chaetigers. Two oblique groups of tentacular filaments arise in two groups on each side of chaetigers 5–6 or 6–7, each with 20–25 tentacles (Fig. 2B).

Notopodium and neuropodium widely separated. Anterior chaetigers with two rows of eight capillary chaetae per fascicle reduced to 3–4 per fascicle posteriorly; 4–5 curved notopodial acicular spines from chaetigers 45–55; 5–6 curved neuropodial acicular spines from chaetigers 34–40; spines with unidentate tips, yellow in color, neuropodial spines larger than notopodial ones (Fig. 2C–F).

Methyl Green Staining Pattern

Distinct staining reaction on prostomium, leaving dorsal region unstained, peristomium staining dorsally and laterally. Body with segmental regions with stain forming complete rings around individual segments, inter-segmental grooves not staining (Fig. 3B); green speckled rings more evident on posterior segments.

Habitat

Intertidal depths in sandy substrate.

Remarks

Cirriformia capixabensis, sp. nov. is most similar to *C. tentaculata* (Montagu, 1808) and *C. pygidia* (Treadwell, 1936) by the distribution of feeding tentacles above chaetigers 5–6. *C. pygidia* is unique in the genus by the absence of notopodial spines, present in all other described species (e.g. Table 1). The early segmental origin of the neuropodial spines in *C. tentaculata* (ch. 25) is the most distinctive feature in comparison with *C. capixabensis*, sp. nov. (ch. 34–40).

The segmental origin of the dorsal tentacles differs in *Cirriformia capixabensis,* sp. nov. from that of *C. chicoi,* sp. nov., with the former having a posterior segmental origin of these tentacles at chaetigers 5–6 or 6–7 while *C. chicoi*, sp. nov. has tentacles from chaetigers 2–3, 3–4 or 4–5. Both species had MGSP forming complete rings on certain the body segments, on *C. chicoi,* sp. nov. the bands are limited to the posterior half of individual segments while in *C. capixabensis,* sp. nov., the entire segment is

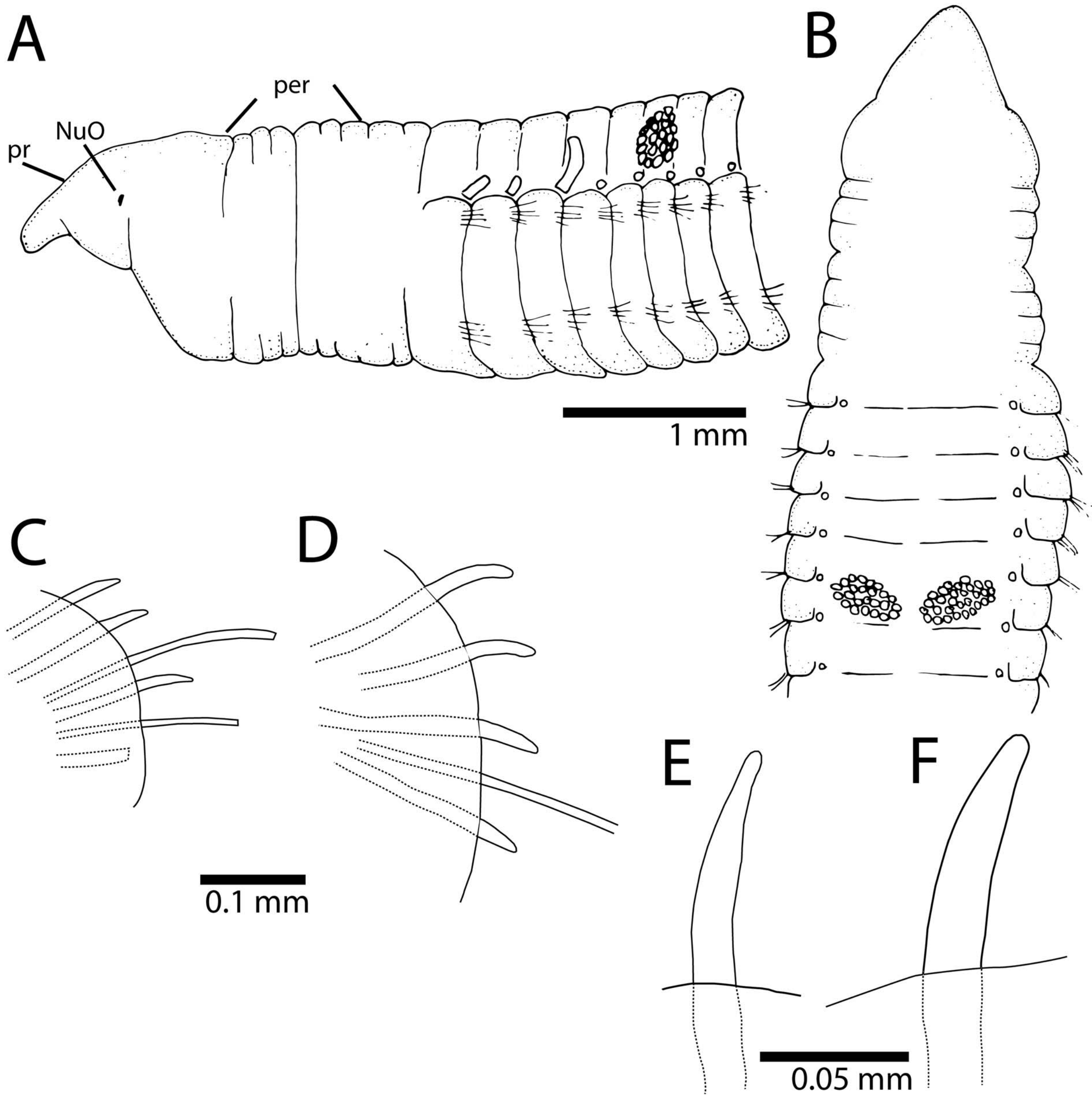

Figure 2. *Cirriformia capixabensis,* sp. nov. A, anterior end in lateral view; B, anterior end in dorsal view; C, notopodium of chaetiger 115; D, neuropodium of chaetiger 115; E, notopodial spine of same chaetiger; F, neuropodial spine of same chaetiger.
doi:10.1371/journal.pone.0112727.g002

stained leaving only the inter-segmental grooves unstained. See Table 1 for comparison with other *Cirriformia* species from the Atlantic Ocean and molecular identity section for a molecular comparison between *Cirriformia capixabensis,* sp. nov. and *C. chicoi,* sp. nov.

Etymology

This species is named after the type locality and the term 'capixaba' refers to those who are born in the Espírito Santo state.

Reproduction

One female was collected with oocytes of about 70 µm in diameter. Asexual reproduction or regenerating individuals were not observed.

Molecular identity

COI fragments of 658 bases pair (bp) were obtained from three individuals, one from Espírito Santo, Vitória, Boi Island and two from Espírito Santo, Guarapari. These sequences were deposited in GenBank under the accession numbers, KM192161-KM192163. The three COI sequences have no differences. See Molecular identity in *C. chicoi,* sp. nov. for inter-specific comparisons between the two *Cirriformia* species.

Distribution

This species is only known from Guarapari and Boi Island (Vitória), both localities in the Espírito Santo state, southern Brazil (Fig. 1).

Cirriformia chicoi, sp. nov. [urn:lsid:zoobank.org:act:2159F014-

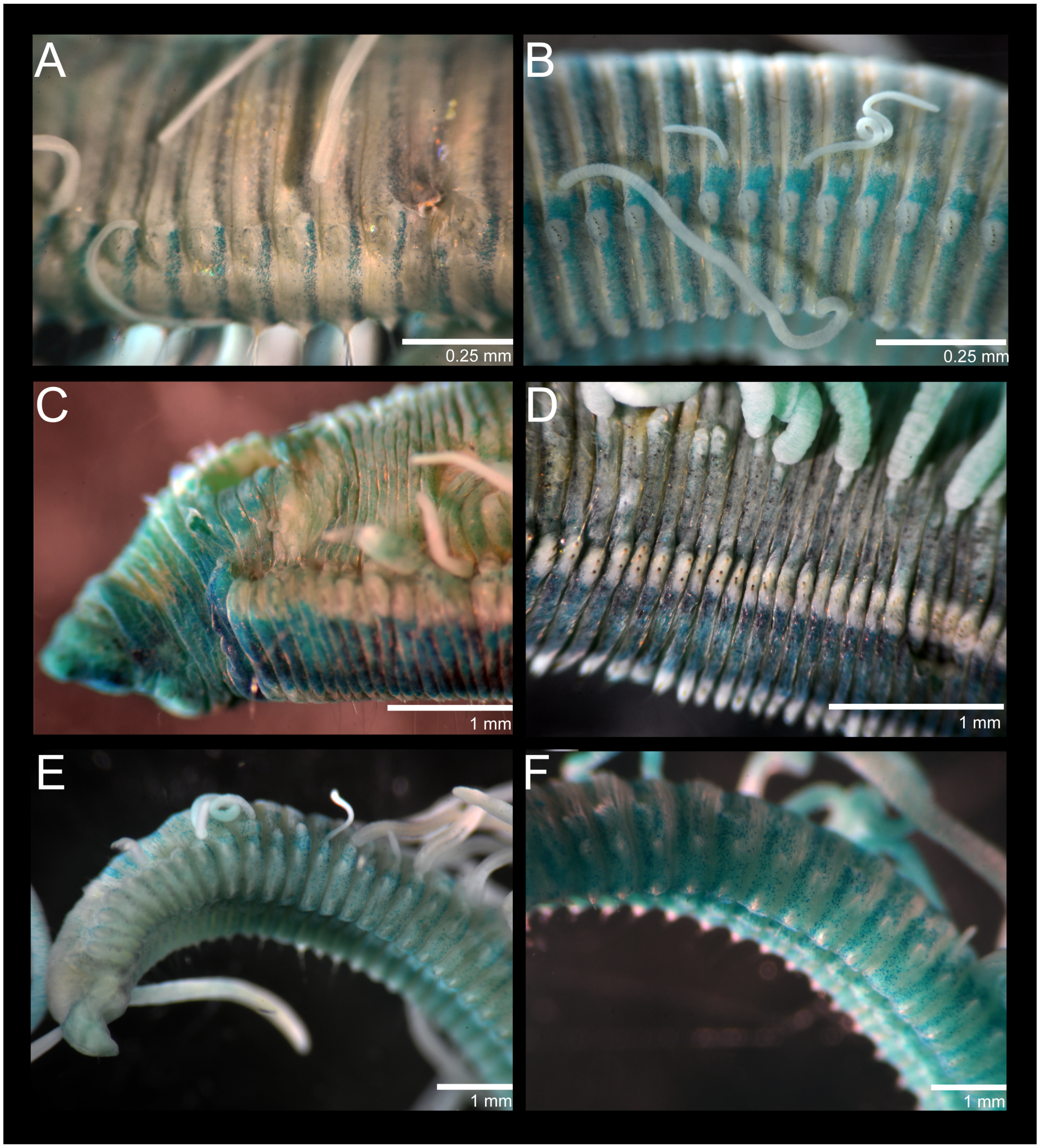

Figure 3. *Cirriformia chicoi,* sp. nov. A, MGSP of mid-body segments. *Cirriformia capixabensis* sp. nov. B, MGSP of mid-body segments *Timarete oculata*. C, anterior end in lateral view showing MGSP; D, mid-body segments in lateral view showing MGSP and insertion of branchial filaments. *Timarete ceciliae* sp. nov. E, anterior end in lateral view showing MGSP; F, mid-body segments in ventral view showing MGSP.
doi:10.1371/journal.pone.0112727.g003

F81C-4B25-A3B8-C4698EF16BF6]
Figures 3 (A), 4 and 5.

Material examined

Type material: Holotype: Brazil: Bahia, Salvador, Itapuã Beach, intertidal pools in *Halimeda opuntia* pillows, 12° 57′ 26.4″ S, 38° 21′ 33.8″ W, July/11/2011, coll. W. Magalhães and L. Martins (IBUFRJ-3781). Paratypes: same locality, date and collector as holotype (6, IBUFRJ-3559).

Non-type material: Brazil: Paraíba, João Pessoa, Cabo Branco Beach, 7° 8′ 40.9″ S, 34° 48′ 31.1″ W, Aug/28/2011, coll. E. Lopes and M. Contins (1 complete, IBUFRJ-3556); Rio de Janeiro, Cabo Frio, Japonês Island, 22° 52′ 49.2″ S, 42° 0′ 19.2″ W, Jun/2013, coll. E.M. Costa-Paiva (1 complete and 1 incomplete, IBUFRJ-3557); Ceará, Fortaleza, unknown date (1 complete and 1 incomplete, IBUFRJ-3558); Espírito Santo, Vitória, Boi Island, 20° 18′ 35.7″ S, 40° 17′ 3.46″ W, Apr/08/2012, coll. B. Silva (1 complete, IBUFRJ-3560).

Table 1. Morphological characteristics of species currently assigned to *Cirriformia* in the Atlantic Ocean.

Species	Type Locality	Prostomium	Peristomium	Origin of branchiae	Feeding tentacles	Capillary chaetae	Notopodial spines	Neuropodial spines	Pygidium	MGSP	References
afer (Ehlers, 1908)	Great Fish Bay, Angola	Short, broadly rounded; eyes absent, nuchal organs not observed	Three same-sized annuli	Ch. 1	Ch. 2; 8–10 tentacles each; large dorsal gap	Anteriorly: two rows of 10–12 each	Fragmented specimens; origin 10–12 chaetigers after origin of neuropodial spines; after chaetiger 60; 2–3 spines	10–12 chaetigers before appearance of notopodial spines; 2–3 spines; similar to notopodial ones; short, slender	Ventral lobe; dorsal anal aperture	Homogeneous staining; branchiae and feeding tentacles with darker stain	Based on specimens (MfN 4483, 6756)
capixabensis sp. nov.	Canto Beach, Espírito Santo, southern Brazil	Conical, as long as 2 anterior chaetigers, eyes absent, large postero-lateral nuchal organs	Elongate, as long as four anterior chaetigers; three similar-sized annuli, sub-annulated	Ch. 1; absent last 20–30 chaetigers	Oblique groups; ch. 5–6 or 6–7; 20–25 tentacles each; small dorsal gap	Anteriorly: two rows of eight each; Posteriorly: 2 rows of 3–4 each	Origin on 45–55; 4–5 spines; unidentate and similar to neuropodial spines	Origin on 34–40; 5–6 spines	Ventral lobe; dorso-terminal anal aperture	Segmental regions stained and forming complete rings, inter-segmental regions not stained	This study
chicoi sp. nov.	Itapuã Beach, Bahia, northern Brazil	Elongate, as long as four anterior chaetigers, eyes absent, discrete postero-lateral nuchal organs	As long as six anterior chaetigers; three annuli, last one slightly longer	Posterior end of peristomium; absent on last third	Oblique groups; ch. 3–4 or 4–5; 18–20 tentacles each; small dorsal gap	Anteriorly: two rows of 7–8 each; Posteriorly: few	Origin on 31–42; 4–5 spines; knobbed-like spines and similar to neuropodial spines	Origin on 19–24; five spines	Small ventral lobe; terminal anal aperture	Mid-body region and posterior end stained with transversal bands on posterior half of segments forming complete rings	This study
capillaris (Verrill, 1900)	Bermuda, north Atlantic	Short, anteriorly rounded; eyes absent	Not described	Not described	Transverse groups; ch. 4; 3 tentacles each	Fewer posteriorly; notopodia with 1–2 capillaries posteriorly while capillaries scarcely seen or absent on neuropodia	Origin unknown; 2–3 spines; nearly straight spines	Origin on 8; three spines; curved and larger than notopodial spines	Not described	Not described	[20,27]
grandis (Verrill, 1873)	New England, north Atlantic	Anteriorly rounded; eyes absent	Not described	Posterior end of peristomium	Ch. 1; 10–12 tentacles each; large dorsal gap	Notopodial capillaries longer than neuropodial ones	Origin on 35; similar to neuropodial ones and up to 2; companion capillaries present	Origin on 19; 1–2 heavy spines and long companion capillary	Not described	Not described	[20]
pygidia (Treadwell, 1936)	Nonsuch island, Bermuda, north Atlantic	Broadly rounded; eyes or nuchal organs not described	Three annuli, second one shorter than first and third	Not described	Ch. 5–6	Present throughout, long, slender	Absent	Origin on 20; dark brown, curved, long	Not described	Not described	[32]

Table 1. Cont.

Species	Type Locality	Prostomium	Peristomium	Origin of branchiae	Feeding tentacles	Capillary chaetae	Notopodial spines	Neuropodial spines	Pygidium	MGSP	References
tentaculata (Montagu, 1808)	South Devonshire, England, Western Europe	Broadly rounded; eyes absent	3.5x as long as prostomium; three annuli	Ch. 1	Ch. 5–6 or 6–7	Long, slender, present throughout	Origin on 50; 3–5 spines	Origin on 25; 4–5 spines	Not described	Not described	Based on Japanese specimens [30] as original description [31] did not contain relevant morphological information
tortugaensis (Augener, 1922)	Bird Key Riff, Tortugas, Southern Florida	Short, rounded distally; eyes or nuchal organs not observed	As long as three anterior chaetigers; three same-sized annuli	Only observed from posterior end of ch. 2; absent on last third	Oblique groups; chaetigers 6–8 or 4–5; small dorsal gap	Anteriorly: two rows of 5–6 each; Mid-body: long natatorial chaetae; Posteriorly: fewer chaetae	Origin on 10 (3–4); unidentate	Origin on two (3–4); thicker and shorter than notopodial spines	Posterior end tapers before pygidium; terminal anal aperture	No staining reaction	Based on syntypes (MfN 6405)

The species *Pentacirrus julianae* Wesenberg-Lung, 1958 described from the Lesser Antilles was not included as it needs to be revised and the type may be a juvenile individual; distribution of feeding tentacles in two rows above several segments resembles those in *Protocirrineris* species although curved spines are present.
doi:10.1371/journal.pone.0112727.t001

Description

Complete specimens 16–38 mm long, 1.5–2.5 mm wide for 117–162 chaetigers. Body elongated, rounded dorsally and flattened ventrally; parapodia form lateral shoulders on thoracic region (Fig. 4A). Color in alcohol white to pale yellow; in life, body yellow, feeding tentacles brick red, with branchial filaments same color as body. Pygidium with ventral lobe and anal aperture placed terminally (Fig. 5E).

Prostomium elongate, as long as four anterior chaetigers, with pair of postero-lateral nuchal organs (Figs 4A, B, 5A). Peristomium with three annulations, as long as six anterior chaetigers; last annulus slightly longer than other two (Figs 4A, B, 5A). Branchial filaments first present on posterior end of third peristominal annulus (Fig. 5B); present in most segments on first one-third of body and absent on last one-third. Two oblique groups of tentacular filaments arise on each side of chaetigers 4–5 (or 3–4 on smaller specimens) with 18–20 (Fig. 4B).

Notopodium and neuropodium widely separated. Anterior chaetigers with two rows of 7–8 capillary chaetae per fascicle reduced to few companion capillaries posteriorly (Fig. 5C); 4–5. curved notopodial acicular spines from chaetigers 31–42 reduced to 3–4 spines posteriorly; 5–6 curved neuropodial acicular spines from chaetigers 19–24, reduced to 4–5 spines posteriorly; spines knobbed-like or sub-dentate (Fig. 5D), all yellow in color; juvenile/ smaller individuals with distinct bidentate spines (Fig. 4C).

Methyl Green Staining Pattern

Anterior end, including prostomium and peristomium, staining lightly. Mid-body region and posterior end stained with transversal bands on posterior half of segments forming complete rings (Fig. 3A); bands more evident on last third of the body.

Habitat

This species was collected associated with the calcareous alga *Halimeda opuntia* on the edges of tidal pools at intertidal depths and on soft bottom sediments.

Remarks

This species is unique in that juvenile individuals had bidentate acicular spines, whereas mature individuals had sub-dentate spines. The development of *C. moorei* (as *C. spirabrancha)* was studied by [14] and all juveniles were found to have bidentate spines; in adults, these spines were unidentate. This has also been reported in *C. tentaculata* [15] and may be universal during the early development of multitentaculates.

Cirriformia chicoi, sp. nov. is most similar to *Cirriformia grandis* (Verrill, 1873) by the peristomial origin of the branchial filaments and segmental origin of spines on notopodia and neuropodia. It differs most noticeably by the segmental origin of the feeding tentacles, on chaetiger 1 in *C. grandis* and on chaetigers 3–4 or 4–5 in *C. chicoi,* sp. nov. and by the shape of the spines being heavy and unidentate in *C. grandis* and slender and knobbed-like in adults of *C. chicoi,* sp. nov. Table 1 shows morphological characteristics of all *Cirriformia* species described from the Atlantic ocean for comparison.

Etymology

This new species is named after Dr. Francisco Barros (a.k.a. Chico), a Brazilian Benthic Ecologist who introduced and motivated the author (WFM) to the study of polychaetes.

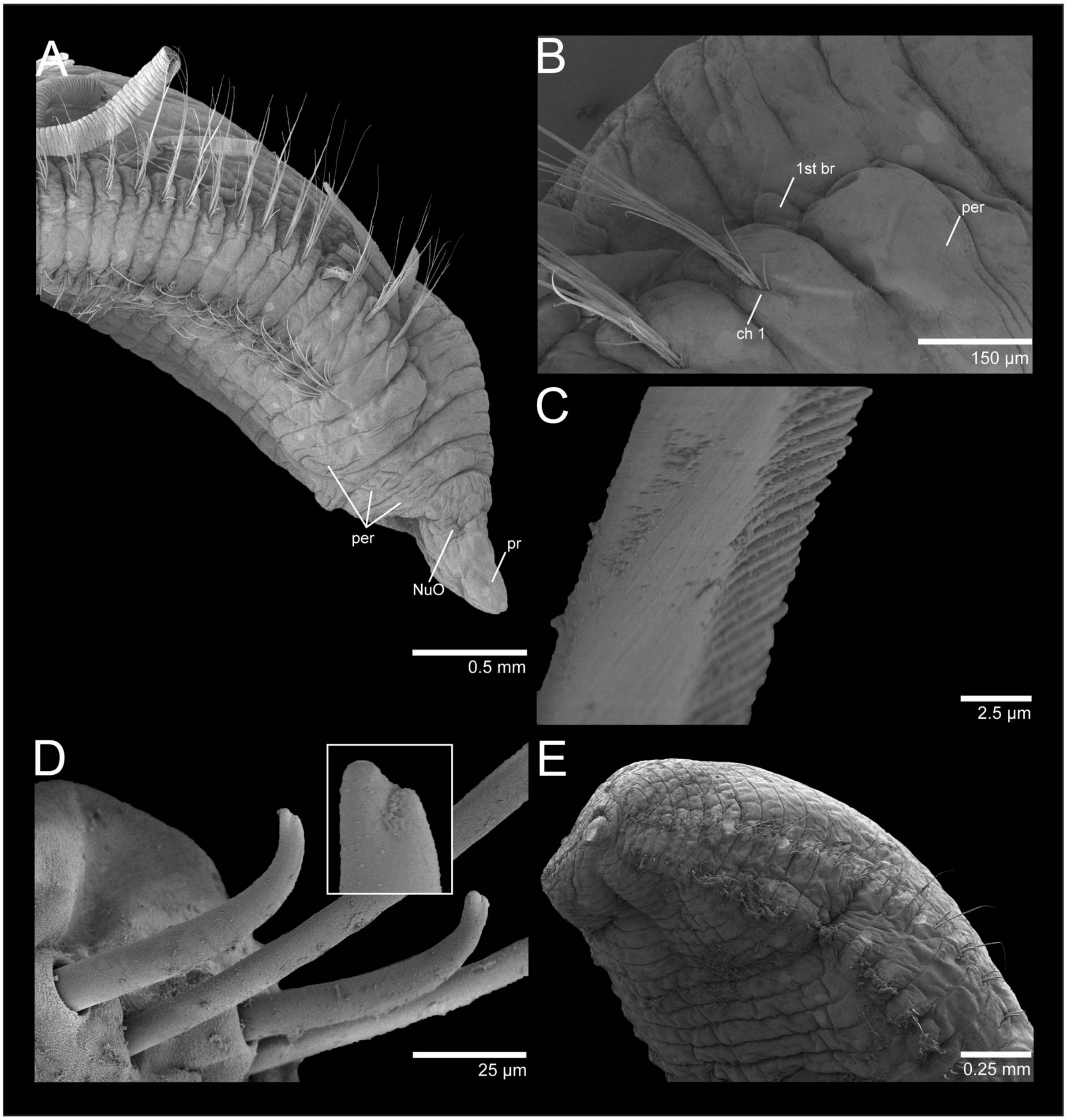

Figure 4. ***Cirriformia chicoi,* sp. nov.** A, anterior end in lateral view; B, anterior end in dorsal view; C, bidentate acicular spines from juvenile individuals.
doi:10.1371/journal.pone.0112727.g004

Reproduction

Sexual reproduction occurs for this species as some females were collected bearing oocytes of 70–80 µm in diameter. Asexual reproduction or regenerating individuals were not observed.

Molecular identity

COI fragments of 658 bp and a 16S fragment of 522 bp were obtained from two and one individual, respectively. All individuals were from Rio de Janeiro, Cabo Frio, Japonês Island. The COI and 16S sequences were deposited in GenBank under the accession numbers, KM192164-KM192165 and KM192189, respectively. The two COI sequences differed by 7 bp (*p*-distance and K2P = 1.1%). The COI inter-specific genetic distance between *C. chicoi,* sp. nov. and *C. capixabensis,* sp. nov. was of 22.1% – 26.3% (*p*-distance - K2P).

Distribution

This species is known from northern and southern Brazil (Fig. 1). The type locality is Itapuã Beach in Salvador, Bahia state.

Timarete Kinberg, 1866
Type Species: Cirratulus anchylochaetus Schmarda, 1861, designated by Hartman, 1959.

Diagnosis (After Blake, 1996): Prostomium wedge-shaped, with or without eyes. Body nearly round in cross section, with segments distinct. Grooved tentacular filaments arising in two

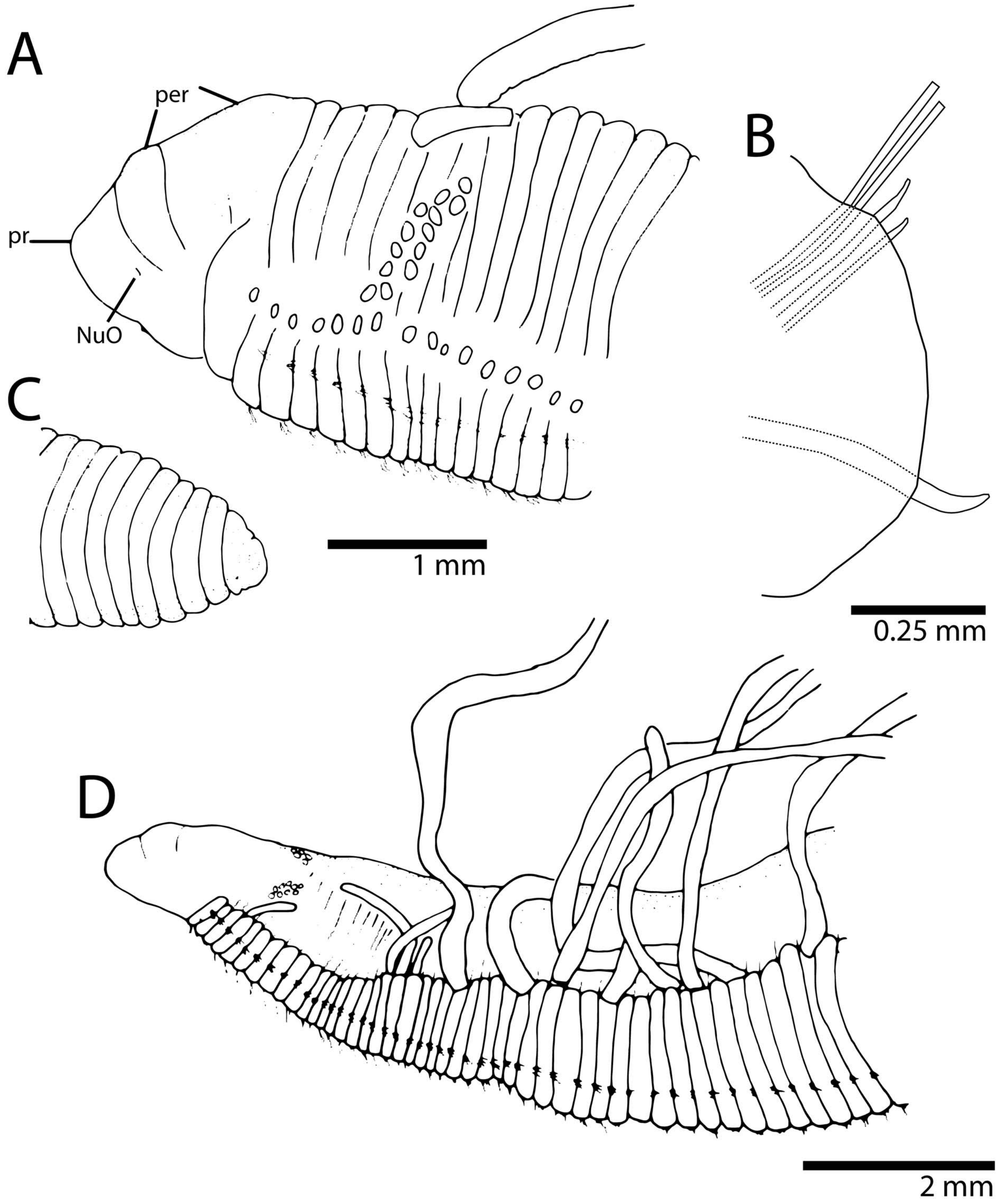

Figure 5. SEM of *Cirriformia chicoi,* sp. nov. A, anterior end in lateral view; B, detail of anterior region showing origin of branchial filaments; C, capillary chaeta; D, subdentate acicular spines; E, posterior end with pygidium.
doi:10.1371/journal.pone.0112727.g005

groups from dorsum of two or more anterior chaetigers, posterior to chaetiger 1. Branchiae occurring singly or with several filaments per parapodium, individual branchial filaments robust, becoming more dorsal in origin in middle body segments, with each sometimes forming dorsolateral bulge over notopodium. Chaetae including capillaries and acicular spines.

Timarete caribous (Grube & Ørsted in Grube, 1859)
Figure 6, 7 (A and B) and 8 (A and B)
Cirrhatulus caribous Grube & Ørsted in Grube, 1859: 106 [16].
Cirriformia caribous; Kirkegaard 1981: 264–265, Fig. 1 [17].
Timarete caribous; Petersen 1999: 116 [18]; Çinar, 2009: 2305–2307, Fig. 7 [19].
Cirratulus melanacanthus (Fr. Müller & Grube in Grube, 1873),

new synonym. *Cirratulus danielsi* Hansen, 1882: synonymized with *C. melanacanthus* by Hartman (1959)

Material examined

Brazil: Santa Catarina, Desterro (now Florianopolis Island), southern Brazil, coll. Grube; syntypes of *Cirratulus melanacanthus* (6, MfN 5677a; 18, MfN 5677b); Rio Grande do Norte, Rocas Atoll, 3° 52′ 1.81" S, 33° 48′ 58.2" W, Oct./23/2009, coll. R. Barroso and S.M.Q. Lima (2, IBUFRJ-3563); Pernambuco, Fernando de Noronha Archipelago, Sueste Beach, 3° 51′ 58.4" S, 32° 25′ 29" W, Aug/03/2013, coll. C. Barboza and G. Mattos (9+4 specimens regenerating anterior end+1 regenerating posterior end, IBUFRJ-3564); Bahia, Abrolhos Archipelago, 17° 57′ 53.8" S, 38° 42′ 24.4" W, coll. R. Barroso (13 complete, IBUFRJ-3565); Bahia, Salvador, Ribeira Beach, 12° 54′ 34.1" S, 38° 29′ 49.2" W, Mar/11/2012, coll. J. Zanol, P.C. Paiva and V.C. Seixas (17 complete, IBUFRJ-3566); São Pedro e São Paulo Archipelago, Enseada Bay, 0° 55′ 2.05" N, 29° 20′ 45" W, May/03/2011, coll. E. Lanna and L. Pessoa (5 incomplete, IBUFRJ-3567).

Colombia: Bay of Cartagena, Manzanillo Island, coll. Ricardo Rojas, approx. 10° 30′ N, 75° 30′ W, Sept. 1977, under roots of *Rhizophora*, less than 50 cm deep, identified as *Cirriformia filigera* by M.H. Pettibone (3, USNM 43506).

U.S.A.: Florida, Safe Harbor, Stock Island (near Key West), Florida Keys, Sta. 3B, 16 feet of water, July/24/1970, coll. Richard Chesher, identified as *Cirriformia filigera* by C.D. Long (9, USNM 57848); Safe Harbor, Stock Island (near Key West), Florida Keys, Sta. 3C, 20 feet of water, July/24/1970, coll. Richard Chesher, identified as *Cirriformia filigera* by C.D. Long (3, USNM 57849); Safe Harbor, Stock Island (near Key West), Florida Keys, Sta. 3A, 6 feet of water, July/24/1970, coll. Richard Chesher, identified as *Cirriformia filigera* by C.D. Long, in calcareous encrustations on wall, very abundant (~50, USNM 57847).

Description

Specimens 9–38 mm long, 1–4 mm wide for 68–192 chaetigers. Body in cross-section, dorsally rounded and ventrally flat; larger specimens (>30 mm) with shallow or deep ventral groove from middle to posterior end. Color in alcohol pale yellow to light brown, ventral part of prostomium, peristomium and first three chaetigers with dark pigmentation; in life, with body, branchiae and feeding tentacles orange to reddish (Fig. 7A); some specimens with darker pigmentation on dorsum and anteriorly on ventrum. Pygidium with small ventral lobe and dorso-terminal anal aperture (Fig. 6C).

Prostomium short, conical with rounded end, twice as wide as long; eyes absent, nuchal organ depression postero-lateral (Fig. 6A). Peristomium also short, as long as two anterior chaetigers with three similar sized annulations; peristomial annuli well-separated in large individuals (Fig. 6A, D). First pair of branchial filaments arise on posterior end of third peristomial annulus; branchiae one pair per segment (but see Remarks) on anterior one-third of body, few on mid-body segments and absent on last one-third. Branchiae shift abruptly to mid-dorsum forming lateral bulge over notopodia (Fig. 6D); segmental origin of branchiae related to size, ranging from chaetigers 10–26 (see Fig. 8B). On largest branchial shift, space in between same segment branchial pairs smaller than space between each filament and notopodial base; last branchial filaments still arise from mid-dorsum, not returning to near notopodial base. Two oblique groups of about 10–16 tentacular filaments arise above two or three chaetigers, e.g. 3–4, 4–5 or 5–7 (Fig. 6A).

Notopodium and neuropodium separated. Anterior chaetigers each with two longitudinal rows of 4–5 capillary chaetae. Notopodial acicular spines similar throughout numbering 3–4 (rarely 5) from chaetigers 15–44 (see Fig. 8A), with alternating capillaries; neuropodial acicular spines of two types: 1) spines similar to those of notopodia, numbering 3–4 from chaetigers 8–24 (see Fig. 8A), mid-body chaetigers these spines begin to enlarge, reaching maximum thickness around chaetigers 35–85; 2) in posterior neuropodia, one (rarely two) large, curved, dark acicular spines occur without companion capillary chaetae (Fig. 6B).

Methyl Green Staining Pattern

No distinctive staining pattern; entire worm stains light green.

Habitat

This species has been found on calcareous encrustations and soft bottom sediments from intertidal depths to shallow subtidal zones.

Remarks

Syntypes of *Timarete caribous* were examined by [19] and they fully agree with the material from the USA east coast, Colombia, and northern and southern Brazil. There is some size-related variation on segmental origin of dorsal tentacles, noto- and neuropodial spines and branchial shift to mid-dorsum. Specimens undergoing asexual reproduction may even increase the variability of these characters. This species is easily distinguishable from other *Timarete* species by the presence of a large, dark and single neuropodial spine on posterior segments and position of branchial filaments on mid-body and posterior segments at a considerable distance from notopodia. The branchial filaments in this species occur as a single pair per segment but some regenerating anterior ends had more than a pair per segment.

Syntypes of *Cirratulus melanacanthus* (Fr. Müller & Grube in Grube, 1873), originally described from Desterro (now Florianópolis Island), southern Brazil, were examined and agree with available descriptions of *Timarete caribous*. *Cirratulus melanacanthus* is herein proposed to be a junior synonym of *T. caribous*. Another multitentaculate cirratulid described from Brazil, *Cirratulus danielsi* Hansen, 1882 had been synonymized with *C. melanacanthus* [1] and should also belong to *T. caribous*.

Reproduction

Many specimens were found with the anterior and/or posterior end in process or completely regenerated (e.g. Fig. 7B). Therefore, asexual reproduction is likely to occur in this species. A female with oocytes measuring 80–100 mm in diameter has been reported in [19].

Molecular identity

COI fragments of 658 pb were obtained from 12 individuals, three of each locality: São Pedro e São Paulo Archipelago, Fernando de Noronha Archipelago, Rocas Atoll and Abrolhos Archipelago. Whereas a 16S fragment of 526 bp were obtained from four individuals, three from Fernando de Noronha Archipelago and one from São Pedro e São Paulo Archipelago. The COI and 16S sequences were deposited in GenBank under the accession numbers, KM192166-KM192177 and KM192190-KM192193, respectively. The COI sequences showed 22 variable sites and the maximum difference between two sequences were 19 bp (SPSPA × AA). The intra-specific genetic distance varied from 0%–2.9% (*p*-distance) and from 0%–3.0% (K2P). The 16S

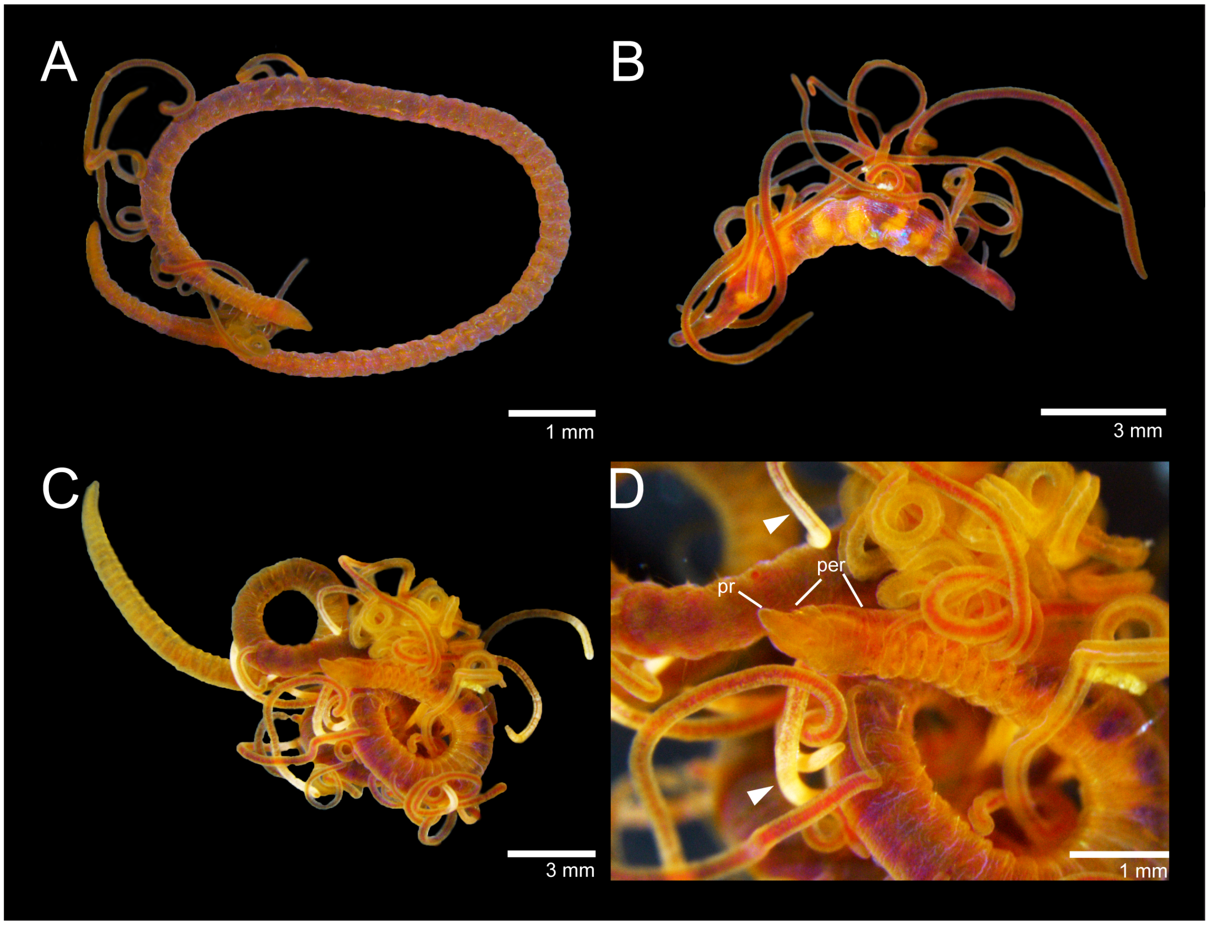

Figure 6. ***Timarete caribous.*** A, anterior end in dorso-lateral view; B, parapodium of chaetiger 60 of a 155 chaetiger individual; C, posterior end with pygidium; D, anterior end in lateral view showing bulge over notopodia formed by shift of branchial filaments dorsally.
doi:10.1371/journal.pone.0112727.g006

sequences showed 7 variable sites and the maximum difference between two sequences were 6 bp (SPSPA × FNA).

The intra-specific genetic distance varied from 0.2%–1.3% (*p*-distance and K2P). The inter-specific genetic distance between *T. caribous* and *T. ceciliae,* sp. nov. was 20.4%–23.9% (*p*-distance - K2P) for COI and 22.0%–26.4% (*p*-distance - K2P) for 16S. For *T. caribous* and *T. punctata* complex the genetic distance was 19.4%–22.6% (*p*-distance - K2P) for COI and 18.0%–20.7% (*p*-distance - K2P) for 16S.

Distribution

The type locality is St. Croix in the Caribbean. This species seems to be widely distributed in the northwestern, central and southwestern Atlantic Ocean. Several specimens from Florida Keys and Colombia, previously identified as *T. filigera,* actually belong to *T. caribous*. In Brazil, this species is widely distributed and has been collected in the northern (Pernambuco and Bahia) and southern states (Rio de Janeiro and Santa Catarina) including oceanic Islands (São Pedro e São Paulo Archipelago, Fernando de Noronha and Rocas Atoll). This species is reported as an invasive species that has entered the Mediterranean through ballast water [19].

Timarete ceciliae, sp. nov. [urn:lsid:zoobank.org:act:A16FD046-C02B-42F0-A5E2-BE32135DB328]
Figures 3 (E and F), 7 (C and D), 8 (C and D) and 9.

Material examined

Type material: Holotype: Brazil, Bahia state, Salvador, Ribeira Beach, 12° 54′ 34.1" S, 38° 29′ 49.2" W, Mar/11/2012, coll. J. Zanol, P.C. Paiva and V.C. Seixas (IBUFRJ-3782). Paratypes: same locality, date and collector as holotype (9, IBUFRJ-3569).

Non-type material: Brazil: Bahia, Salvador, Ribeira Beach, 12° 54′ 34.1" S, 38° 29′ 49.2" W, Mar/11/2012, coll. J. Zanol, P.C. Paiva and V.C. Seixas (51 complete + several incomplete and regenerating, IBUFRJ-3570). Pernambuco, Fernando de Noronha Archipelago, Sueste Beach, 3° 51′ 58.4" S, 32° 25′ 29" W, Aug/03/2013, coll. C. Barboza and G. Mattos (6 incomplete, 3 with anterior or posterior ends in regeneration+13 complete, IBUFRJ-3571).

Description

Holotype 35 mm long, 1.8 mm wide for 165 chaetigers. Paratypes ranging from 9–34.5 long, 0.6–2 mm wide for 94–165 chaetigers. Body in cross-section, dorsally rounded and ventrally flattened with a ventral groove formed by projection of parapodial shoulders. Color in alcohol pale yellow or light grey; some individuals with ventral pigmentation; tentacles and branchiae slightly lighter than body; color in life, yellowish to light orange with branchiae lighter than body and feeding tentacles, some branchiae with whitish areas (Fig. 7C, D). Pygidium with either

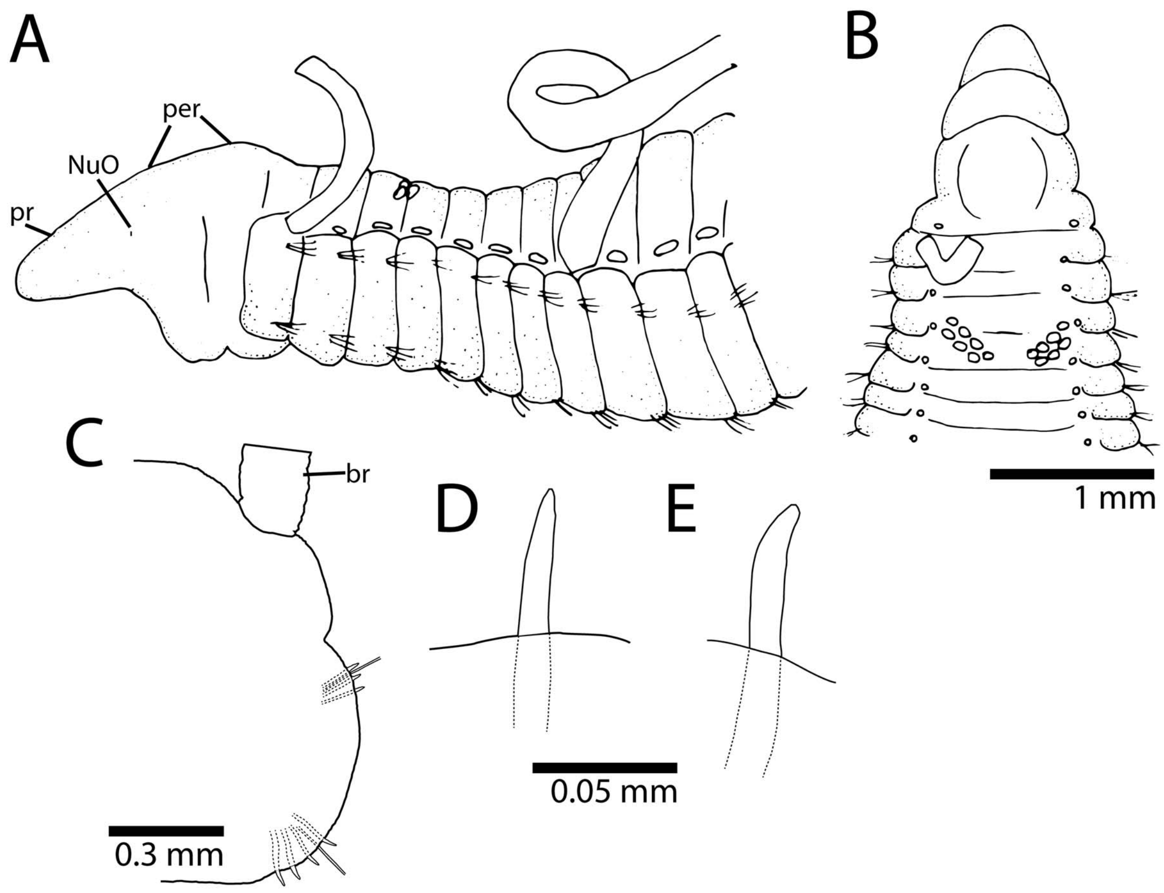

Figure 7. ***Timarete caribous.*** A, complete live specimen; B, live specimen undergoing regeneration of both anterior and posterior ends. *Timarete ceciliae* sp. nov. C, complete live specimen; D, detail of anterior end, white arrows show white regions of branchiae.
doi:10.1371/journal.pone.0112727.g007

terminal anus or small ventral lip with dorso-terminal anal aperture.

Prostomium conical, rounded anteriorly, as long as two anterior chaetigers (Figs 7C; 9A, B); eyes absent, nuchal organ depression postero-lateral. Peristomium as long as four anterior chaetigers, with three similar-sized annulations; first peristomial annulus dorsally inflated forming crest (Fig. 9B). First pair of branchial filaments arise on posterior end of third peristomial annulus; branchiae one pair per segment on anterior one-third of body, scarce on mid-body segments and absent on last one-third of body. Branchiae shift gradually to mid-dorsum from chaetigers 8–14 (Fig. 8D), not forming lateral bulge over notopodia; branchiae on posterior segments with distance from notopodial base similar to distance between noto- and neuropodia. Two slightly oblique groups of 5–6tentacles, arise as early as chaetigers 2–3 but larger specimens with tentacles on chaetigers 4–5 (Fig. 9B).

Notopodium and neuropodium widely separated. Anterior chaetigers with two longitudinal rows of 4–5 capillary chaetae in each row. Notopodial acicular spines similar throughout numbering 2–3 (rarely 4) per fascicle, from chaetigers 11–23 (Figs 8C; 9C, D), with alternating capillaries, reduced to two spines posteriorly. Neuropodial acicular spines similar throughout number 3–4 present from chaetigers 5–12 (Figs 8C; 9C, E), alternated by capillaries; reduced to 2–3 spines posteriorly. Neuropodial acicular spines slightly longer and thicker than notopodial spines, all curved and yellow in color (Fig. 9E, F).

Methyl Green Staining Pattern

Larger specimens with distinct staining reaction (Fig. 3E). Dense green speckles present on individual body segments forming complete rings (Fig. 3F). Branchial filaments also stained with green speckles. Pygidium stained with uniform green.

Habitat

This species is found abundantly in muddy sand in intertidal regions of Ribeira Beach, northern Brazil.

Remarks

Timarete ceciliae, sp. nov. has been collected together with specimens of *T. punctata* complex and *T. caribous*. It differs from species of the *T. punctata* complex by the absence of body pigmentation and from *T. caribous* by the segmental origin of feeding tentacles, branchial shift, and presence of 2–3 neuropodial spine on posterior segments. *T. caribous* is unique by the presence in posterior segments of single heavy spines and absence of companion capillaries.

T. ceciliae, sp. nov. is most similar to *T. hawaiensis* (Hartman, 1956) and to material examined of *T.* nr. *filigera* (Delle Chiaje, 1828) from the Mediterranean by the presence of feeding tentacles above two anterior segments, usually chaetigers 3–4. The segmental origin of spines in notopodia and neuropodia is also overlapping but spines in *T. ceciliae*, sp. nov. appear several segments before than in both *T. hawaiensis* and *T.* nr. *filigera* (see

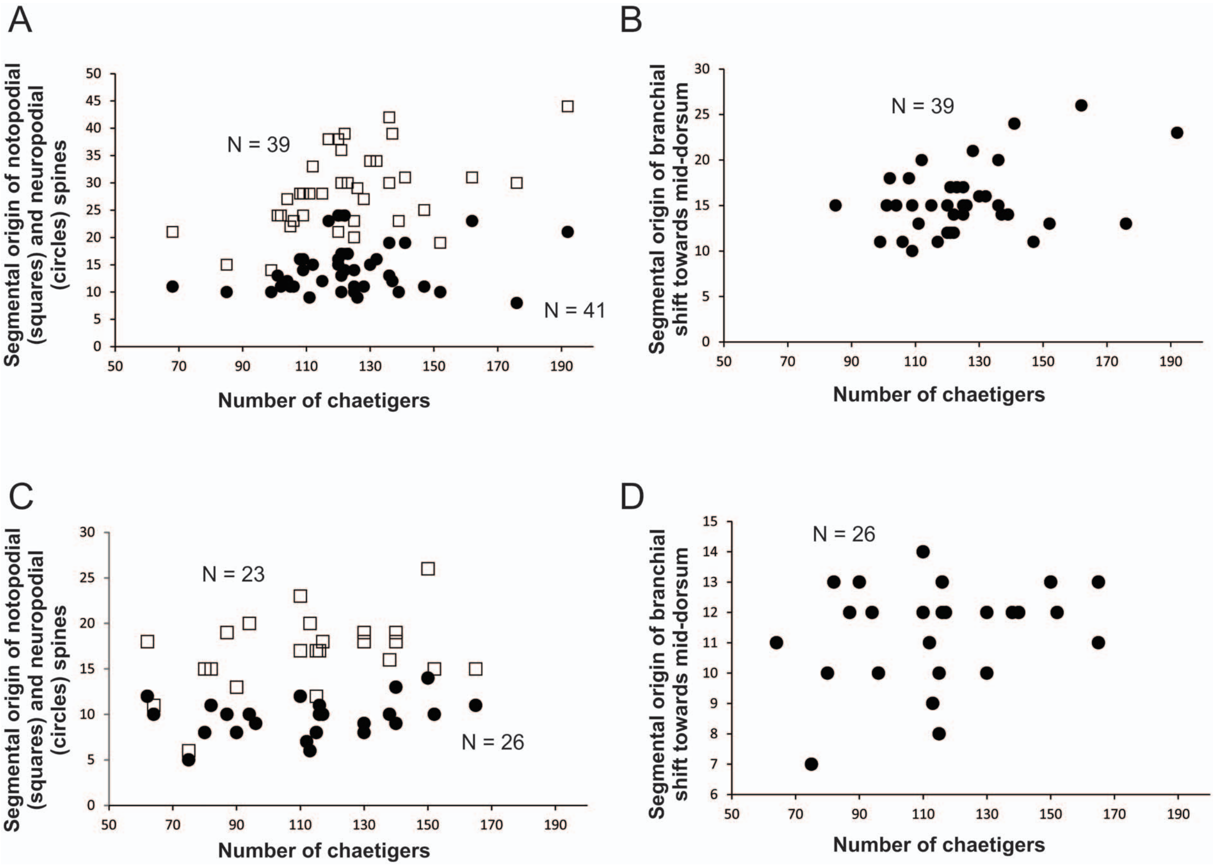

Figure 8. Variation of size-dependent characters in *Timarete caribous:* A, Relationship between the total number of chaetigers and the segmental origin of notopodial and neuropodial spines; B, Relationship between the total number of chaetigers and the segmental origin of the branchial shift towards mid-dorsum. Variation of size-dependent characters in *Timarete ceciliae,* sp. nov.*:* C, Relationship between the total number of chaetigers and the segmental origin of notopodial and neuropodial spines; D, Relationship between the total number of chaetigers and the segmental origin of the branchial shift towards mid-dorsum.
doi:10.1371/journal.pone.0112727.g008

Table 2). The main distinctive feature is the abrupt shift in the position of branchial filaments toward mid-body segments in *T. hawaiensis* and *T.* nr. *filigera* and gradual shift in *T. ceciliae*, sp. nov. More distinctive morphological features can be found in Table 2.

Etymology

This species is named in honor of Dr. A. Cecília Amaral from the Universidade de Campinas, São Paulo, Brazil, for her great contributions to the taxonomy of polychaetes and benthic ecology in Brazil.

Reproduction

Several individuals *Timarete ceciliae*, sp. nov. were collected regenerating either the anterior and/or posterior ends. Regenerating ends were always lighter in color than the rest of the body. Individuals with a regenerating anterior end may have acicular hooks starting on the segment of fission in both noto- and neuropodia. These individuals were excluded from the data recorded for Fig. 8. One individual had a fully regenerated prostomium and peristomium, feeding tentacles and first pair of branchiae, and noto- and neuropodial acicular spines from chaetiger 1.

Molecular identity

COI fragments of 658 bp and 16S fragments of 515 bp were obtained from two and three individuals, respectively. All individuals were from Pernambuco, Fernando de Noronha Archipelago, Sueste Beach and Bahia, Salvador, Ribeira Beach. The COI and 16S sequences were deposited in GenBank under the accession numbers, KM192178–KM192179 and KM192194–KM192196, respectively. The two COI sequences have no differences. The 16S sequences showed one variable site, and the genetic distance between sequences varied from 0% to 0.2% (*p*-distance and K2P). The inter-specific genetic distance between *T. ceciliae* and *T. punctata* complex was 22.2%–26.5% (*p*-distance - K2P) for COI and 19.8%–23.2% (*p*-distance - K2P) for 16S. See Molecular identity in *T. caribous* for an inter-specific comparison between the *T. ceciliae* and *T. caribous*.

Distribution

Type locality is Ribeira Beach in Salvador, Bahia, northern Brazil. This species was also collected further south in the Espírito Santo state.

Timarete oculata (Treadwell, 1932), comb. nov. Figures 3 (C and D) and 10

Audouinia oculata Treadwell, 1932 [7].

Cirratulus flavescens Grube, 1872, not *Cirratulus flavescens*

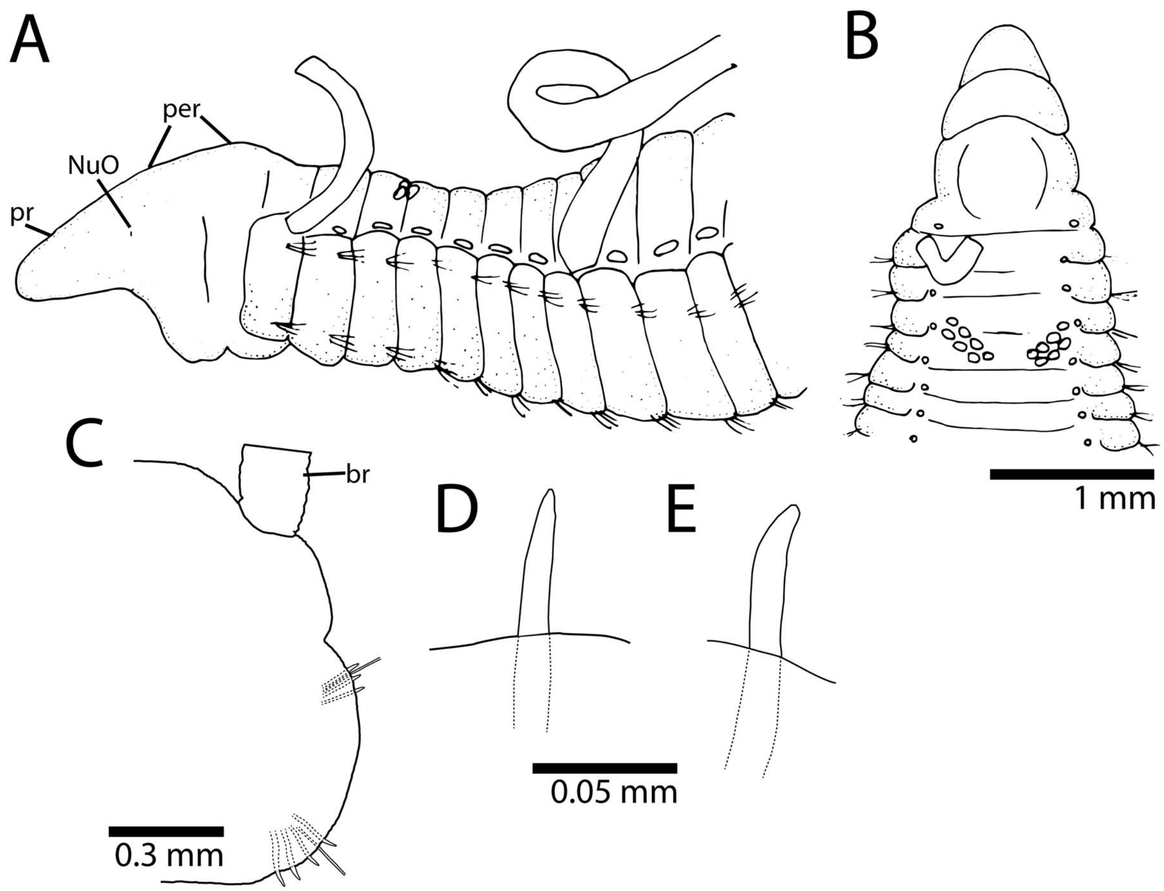

Figure 9. ***Timarete ceciliae,*** **sp. nov.** A, anterior end in lateral view; B, anterior end in dorsal view; C, parapodium of chaetiger 60; D, notopodial spine of chaetiger 60; E, neuropodial spine of same chaetiger.
doi:10.1371/journal.pone.0112727.g009

Johnston, 1825.
Cirriformia filigera; of authors, Hartman, 1942: 127–128 [20]; Amaral *et al*., 2006 (in part) [8].
Timarete filigera; of authors, see Amaral *et al*., 2006 (in part) [8].

Material examined

Type material: Brazil, Villa Bella, São Sebastião Island, São Paulo, holotype (USNM 19640), paratype (1, USNM 19635). *Cirratulus flavescens* Grube, 1872, holotype, from Desterro (now Florianopolis Island), coll. Grube/Fr. Müllers (MfN Q. 4529).

Non-type material: Brazil, Bahia, Abrolhos Archipelago, 17° 57′ 53.8" S, 38° 42′ 24.4" W coll. R. Barroso (1 complete, 3 anterior fragments, IBUFRJ-3568).

Comparative material: *Timarete* cf. *filigera* (Delle Chiaje, 1828): Tunisia, Gulf of Tunis, LeBoc, March 14, 1967, coll. DiGenova by foot, Ref. No. 5, Mediterranean Sorting Center, identified as *Audouinia filigera* by DiGenova, container had a mix of *Cirriformia* and *Timarete* species (4, USNM 39707); *Audouinia filigera nesophila* Chamberlin, 1919: Easter Island, Dec. 20, 1904, collected from shore, identified by R.V Chamberlin (USNM 19758); *Timarete hawaiensis* (Hartman, 1956): Hawaii, Oahu, Hilton Hawaiian Lagoon, Mar/2014, coll. W. Magalhães, several specimens.

Description

Holotype missing pygidium, 110 mm long, 7 mm wide for about 310 chaetigers. Paratype complete with pygidium, 85 mm long, 5 mm wide for about 300 chaetigers. Color in alcohol pale yellow, tentacles and branchiae slightly lighter than body. Pygidium with terminal anus in paratype. In cross-section, body dorsally rounded, ventrally flattened with shallow ventral groove.

Prostomium short, rounded anteriorly, as long as two anterior chaetigers (Fig. 10A); eyes absent, nuchal organ depression postero-lateral. Peristomium as long as 6–8 anterior chaetigers, with three equivalent annulations; peristomial annuli with dorsal sub-annulations (Figs 3C; 10B). First pair of branchial filaments arises on posterior end of peristomium; branchiae one pair per segment on anterior one-third of body, few on mid-body segments and last one-third of body, with about one filament every 10 segments. Branchiae shifted gradually to mid-dorsum from chaetigers 20–25 on holotype and paratype, not forming lateral bulge over notopodia (Fig. 3D); branchiae on very posterior end still arise well above notopodial base. Two oblique groups of about 15–20 tentacular filaments arise on chaetigers 4–5 or 6–7 (Fig. 10B).

Notopodium and neuropodium widely separated. Anterior chaetigers with two longitudinal rows of 8–10 capillary chaetae in each row. Notopodial acicular spines similar throughout numbering 3–4 from chaetigers 58 on holotype and chaetiger 57 on paratype, with spines alternating with capillaries; reduced to two spines posteriorly. Neuropodial acicular spines similar throughout, 3–4 from chaetiger 40 on holotype and chaetiger 38 in paratype, alternating with capillaries; reduced to 1–2 spines posteriorly. Neuropodial acicular spines darker, thicker than

Table 2. Morphological characteristics of species currently assigned to *Timarete*.

Species	Type Locality	Prostomium	Peristomium	Origin of branchiae	Shift of branchiae	Number of branchiae per segment	Feeding tentacles	Notopodial spines	Neuropodial spines	Pygidium	References
caribous (Grube & Ørsted in Grube, 1859)	St. Croix, Caribbean	Short, conical with rounded end, twice wider than long; eyes absent, nuchal organ postero-lateral	Short, as long as two anterior chaetigers with three same-sized annulations	Peristomium	Abrupt; 10–26; forming lateral bulge over notopodia	One pair per segment; exception to few regenerating specimens with more than one pair per segment	Oblique groups; 10–16 tentacles each; 3–4, 4–5 or 5–7; small dorsal gap	Origin on 15–44; 3–4 spines; similar throughout	Origin on 8–24; similar to notopodial spines anteriorly and from chaetigers 35–85, spines become thick, curved, solitary and darker	Small ventral lobe; dorso-terminal anal aperture	This study; [19]
ceciliae sp. nov.	Ribeira Beach, Bahia, northern Brazil	Conical, rounded anteriorly and as long as two anterior chaetigers; eyes absent, nuchal organ postero-lateral	As long as four anterior chaetigers with three same-sized annulations; first peristomial annulus inflated	Peristomium	Gradual; 8–14; not forming lateral bulge	One pair per segment	Slightly oblique; 5–6 tentacles each; 2–3, 2–4 or 4–5; small dorsal gap	Origin on 11–23; 2–3 spines	Origin on 5–12; 2–3 spines; slightly longer and thicker than notopodial ones	Anus terminal or dorso-terminal with small ventral lip	This study
nr. filigera (Delle Chiaje, 1828)	Specimens from Tunisia, Mediterranean Sea (USNM 39707)	Short, rounded anteriorly and as long as three anterior chaetigers; eyes absent, nuchal organ not observed	As long as 6–7 anterior chaetigers, with at least three annulations	Peristomium	Abrupt; 15–20; forming bulge over notopodia	One pair per segment	Oblique; 15–20 tentacles each; 2–5, most filaments above 3–4	Origin on 27–31; 2–3; straight	Origin on 15–21; 4–5(6); darker and thicker than notospines; curved	Small ventral lip and dorsal anal aperture	Based on voucher specimens from the Mediterranean (USNM 39707)
hawaiensis (Hartman, 1956)	Pearl Harbor, Hawaii	Short, broadly rounded; eyes absent, nuchal organ postero-lateral	As long as four anterior chaetigers; three same-sized annulations; first annulus forming a crest	Peristomium	Abrupt; 10–18; forming a bulge over notopodia	One pair per segment	Transverse; 7–9 filaments each; 3–4; wide dorsal gap	Origin on 21–78; 2–3; pale yellow, straight	Origin on 8–19; 3–4; curved and more robust than notopodial spines	Terminal anus	[28]
luxuriosa (Moore, 1904)	Southern California	Triangular, very broad, wider than long; eyes absent	Short, about twice as long as prostomium; one large and three smaller annuli	Chaetiger 1	Gradual; about chaetiger 35	One pair per segment	Slightly oblique; 12 or more tentacles each; 5–6; very small dorsal gap	Origin on 50; 3; pale brown	Origin on 31; 3–4; dark brown, thicker than notopodial ones	Flattened lobe bellow terminal anus	[29]
nasuta Ehlers, 1897	Patagonia, Argentina	Short, triangular, as long as two anterior chaetigers; eyes absent, nuchal organ postero-lateral, rounded	As long as three anterior chaetigers with three annuli; second annulus with a dorsal sub-annulation	Peristomium	Gradual; 12–14; forming bulge over notopodia	One pair per segment	Transverse groups; 15–20 tentacles each; 4–5; small dorsal gap	Origin on 20–30; 3–4 spines; slender than neuropodial spines	Origin on 14–16; 5–6	Ventral lip and dorsal anal aperture	Based on voucher specimens from Argentina (USNM 60690)

Table 2. Cont.

Species	Type Locality	Prostomium	Peristomium	Origin of branchiae	Shift of branchiae	Number of branchiae per segment	Feeding tentacles	Notopodial spines	Neuropodial spines	Pygidium	References
oculata (Treadwell, 1932)	São Sebastião Island, São Paulo, Southern Brazil	Short, rounded anteriorly and as long as two anterior chaetigers; eyes absent, nuchal organ postero-lateral	As long as 6–8 anterior chaetigers, with three same-sized annulations; dorsal sub-annulations present	Peristomium	Gradual; 20–25; not forming bulge over notopodia	One pair per segment	Oblique groups; 15–20 tentacles each; 4–5 or 6–7; small dorsal gap	Origin on 57–58; 3–4; straight	Origin on 38–40; 3–4; darker and thicker than notopodial spines and slightly curved	Terminal anus	This study
perbranchiata (Chamberlin, 1918)	Central California	Broad, truncate; eyes absent	Short with three annulations	Chaetiger 1	Gradual; 11–12; not forming a bulge over notopodia	2–3(4) pairs per segment increasing to 3–5 on post-tentacular segments	Oblique groups; up to 40 tentacles each; 5–7; wide dorsal gap	Origin on 31–35; 2–4 pale spines	Origin on 17–20; 3–4 heavy spines; from chaetigers 55–70, single large, heavy, curved black spines	Flattened lobe below terminal anus	[29]
punctata (Grube, 1859)	St Croix, US Virgin Islands	Short, rounded anteriorly and as long as one or two anterior chaetigers; eyes absent, nuchal organ depression postero-lateral	Short, as long as 2–3 anterior chaetigers, with three same-sized annulations; second and third peristomial annuli with dorsal and ventral sub-annulations	Peristomium (this study)/ chaetiger 1 (Çinar, 2007)	Gradual; 7–18 (this study), 10–26 [24]; forming or not a bulge over notopodia	One pair per segment	Oblique groups; 5 tentacles each (Çinar, 2007), 10–14 tentacles each (this study); 3–4 or 4–5; small dorsal gap	Origin on 8 (Çinar, 2007), 9–25 (this study); 3–4; similar to neuropodial spines	Origin on 6 (Çinar, 2007), 6–17; 3–4	Ventral lip and dorsal anal aperture	This study; [24]

The species *Timarete anchylochaeta* (Schmarda, 1861) from New Zealand and *Timarete japonica* Zachs, 1933 from Japan were not included by the lack of detailed morphological information in the original and subsequent descriptions.

doi:10.1371/journal.pone.0112727.t002

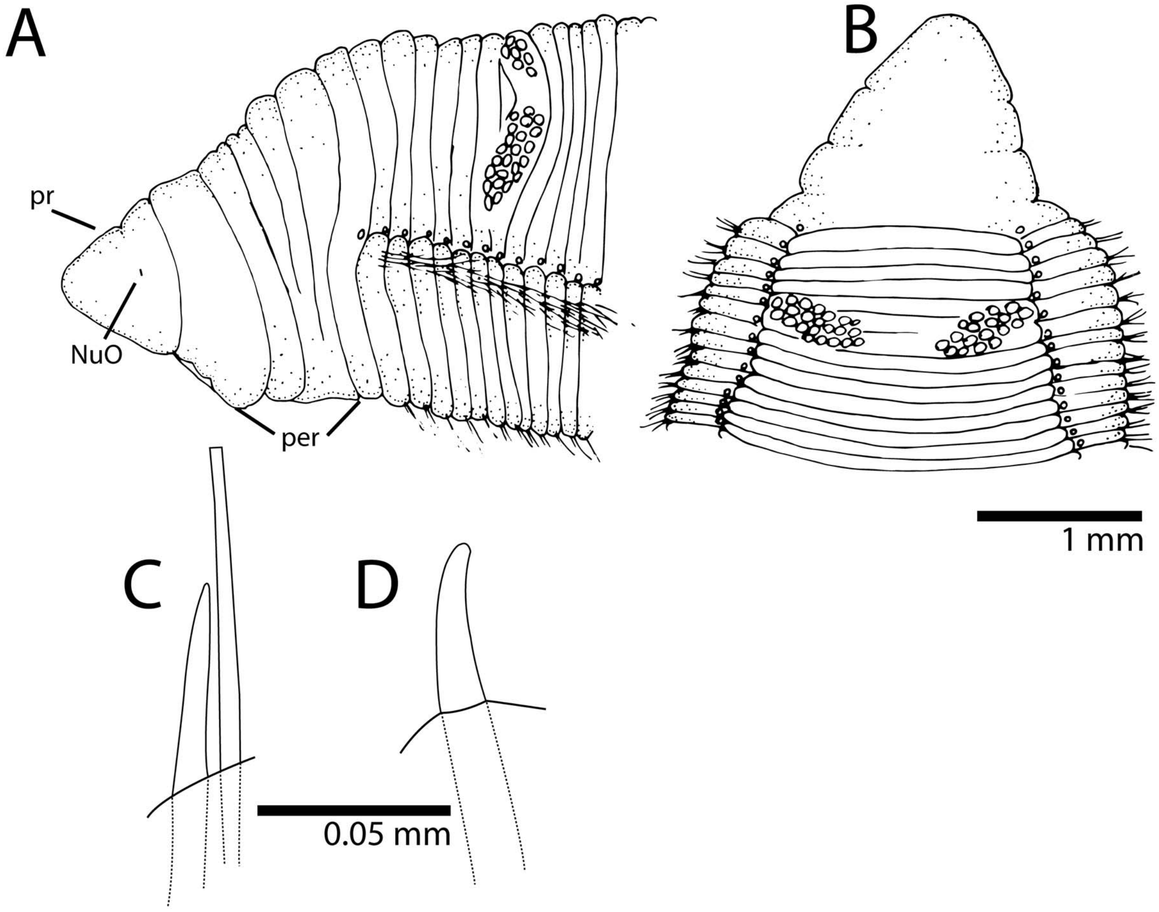

Figure 10. ***Timarete oculata*****.** A, anterior end in lateral view; B, anterior end in dorsal view; C, notopodial spine of chaetiger 55; D, neuropodial spine of chaetiger 55.
doi:10.1371/journal.pone.0112727.g010

notopodial spines; neuropodial spines slightly curved, notopodial spines straight (Fig. 10C, D).

Methyl Green Staining Pattern

Prostomium and peristomium stained with dark green (Fig. 3C). Body staining lightly with green speckles. Parapodial regions staining with dark green except for notopodial and neuropodial ridges (Fig. 3D).

Habitat

Intertidal and shallow subtidal depths.

Remarks

This species is herein considered as valid and transferred to the genus *Timarete* because the branchial filaments shift to mid-dorsum of body although not forming a lateral bulge over notopodia. *Audouinia oculata* was considered a synonym of *T. filigera* [20]. Type material of *T. filigera* is considered to be lost but comparative material from the Mediterranean identified as the latter was examined and shows several morphological differences.

Table 2 shows several morphological characters for *T. oculata,* comb. nov. and its congeners. For instance, the Brazilian species *T. oculata* differs from *T. filigera* by the following characteristics: 1) branchiae shift abruptly to mid-dorsum from chaetigers 15–20 forming a bulge over notopodia in *T. filigera* and gradually from chaetigers 20–25 not forming a bulge over notopodia in *T. oculata*; 2) tentacular filaments arise on chaetigers 3–4 in *T. filigera* and 4–5 in *T. oculata*; 3) notopodial acicular spines start from chaetigers 27–31 in *T. filigera* and from chaetigers 57–58 in *T. oculata*; 4) neuropodial acicular spines start from chaetigers 15–21 in *T. filigera* and 38–40 in *T. oculata*; and. 5) the pygidium has a dorsal anal aperture with a small ventral lip in *T. filigera* and terminal anus in *T. oculata*.

The holotype of *Cirratulus flavescens* Grube 1872 described from Brazil was considered a homonym by [21] probably because the name was preoccupied by *C. flavescens* Johnston, 1825. This holotype was examined and it belongs to *Timarete oculata* comb. nov. The specimen is complete and well-preserved measuring 82 mm long, 3 mm wide for about 300 chaetigers. The prostomium is short and rounded; the peristomium has three annulations, with the third annulus twice as long as the first annuli. Branchial filaments arise from the posterior end of the third peristomial annulus and are absent on the last 50 chaetigers. Branchiae gradually shift to the mid dorsum from about chaetiger 50. Neuropodial hooks occur from chaetiger 48 and notopodial hooks from chaetiger 75.

Reproduction

Holotype and paratype were females and coelom was filled with eggs.

Distribution

The type locality is in São Sebastião Island but this species was also collected in the Abrolhos Archipelago, northern Brazil (Fig. 1). Several records for the occurrence of *Cirriformia filigera* and *Timarete filigera* in Brazil were put together in [8] but it could actually belong to *T. oculata,* comb. nov.

Timarete punctata (Grube, 1859) species complex
Cirrhatulus punctata Grube, 1859: 107 [16].
Cirrhatulus nigromaculatus Grube, 1869: 24–25 [22].
Cirriformia punctata; Hartman, 1956: 292 [23].
Timarete punctata; Petersen, 1999: 116 [18]; Çinar, 2007 (and references therein): p. 755–764, figs. 2–5 [24].

Material examined

Brazil: São Pedro e São Paulo Archipelago, Enseada, 0° 55′ 2.05" N, 29° 20′ 45" W, May/03/2011, coll. E. Lanna and L. Pessoa (30, IBUFRJ-3572); São Pedro e São Paulo Archipelago, Enseada, 0° 55′ 2.05" N, 29° 20′ 45" W, Jun/18/2011, coll. G. Rodriguez and F. Azevedo (6, IBUFRJ-3573); Rio Grande do Norte, Rocas Atoll, 3° 52′ 1.81" S, 33° 48′ 58.2" W, Oct/22/2009, coll. R. Barroso and S.M.Q. Lima (17, IBUFRJ-3574); Rio Grande do Norte, Rocas Atoll, 3° 52′ 1.81" S, 33° 48′ 58.2" W, Jan./01/2012, coll. V.C. Seixas and C.C. Paiva (5, IBUFRJ-3575); Bahia, Salvador, Ribeira Beach, 12° 54′ 34.1" S, 38° 29′ 49.2" W, Mar/11/2012, coll. J. Zanol, P.C. Paiva and V.C. Seixas (3, IBUFRJ-3576); Bahia, Salvador, Itapuã Beach, intertidal rocky pools in *Halimeda opuntia* pillows, July/11/2011, coll. W. Magalhães and L. Martins (6 complete and 7 showing regenerating either anterior or posterior ends).

Description

Specimens 1.5–21 mm long, 0.2–2 mm wide for 30–156 chaetigers. Color of living specimens dark yellow with black pigment spots throughout body and branchial filaments; dorsal tentacles with dark rings. Preserved specimens with dark grey body; pigment retained on branchiae and dorsal tentacles. Body in cross section, dorsally rounded and ventrally flat without ventral groove. Pygidium with ventral lip and dorsal anal aperture.

Prostomium short, rounded anteriorly, as long as one or two anterior chaetigers; eyes absent, nuchal organ depression postero-lateral. Peristomium as long as 2–3 anterior chaetigers, with three similar sized annulations; second and third peristomial annuli with dorsal and ventral sub-annulations. First pair of branchial filaments arise on posterior end of third peristomial annulus; branchiae with one pair per segment on anterior one-third of body, few on mid-body segments and absent on last 10–20 segments. Branchiae shift gradually towards mid-dorsum but not reaching middle region, from chaetigers 7–18, forming lateral bulge over notopodia, bulge sometimes not observed; branchiae on very posterior end arise from a small distance above notopodial base. Two oblique groups of about 10–14 tentacular filaments arise on chaetigers 3–4 or 4–5.

Notopodium and neuropodium widely separated. Anterior chaetigers with two longitudinal rows of five capillary chaetae in each row. Notopodial acicular spines similar throughout numbering 3–4 from chaetigers 9–25, with alternating capillaries, reduced to 1–2 spines posteriorly. Neuropodial acicular spines similar throughout number 3–4 present from chaetigers 6–17, alternated by capillaries; reduced to 1–2 spines posteriorly. Neuropodial and notopodial acicular spines similar, short and slightly curved distally.

Methyl Green Staining Pattern

No distinctive staining pattern; entire worm stained with light green.

Habitat

This species was collected associated with the calcareous alga *Halimeda opuntia* on the edges of tidal pools and on sandy and muddy sediments.

Remarks

These specimens of *Timarete punctata* are similar to the redescription of the type material by [24] but some variation on body morphology and coloration pattern was found. Juvenile specimens with elongated black spots over the body and mature specimens with rounded black spots on a dark brownish body were described in [24]. This author also observed densely pigmented branchial filaments and tentacular filaments with black lateral stripes. The material collected in Brazil is believed to belong to at least two different species based on pigmentation of live and preserved specimens and on body morphology. However, there is a need for molecular evidence to help distinguish the suspected cryptic species within the *T. punctata* species complex. Since it is beyond the scope of this study, we designate the species collected along the Brazilian coast as *T. punctata* species complex until further integrated studies are performed.

Reproduction

Some specimens were found with anterior and/or posterior end in process or completely regenerated. Therefore, asexual reproduction is likely to occur in this species as also reported by [18]. Juvenile individuals (30–40 chaetigers) were also collected indicating the occurrence of sexual reproduction. One specimen from the Mediterranean was collected with oocytes in the coelom having a mean diameter of 65.5 μm [24].

Molecular identity

COI fragments of 633 bp and 16S fragments of 533 bp were obtained from 12 individuals, three of each locality: São Pedro e São Paulo Archipelago, Rocas Atoll and Bahia, Salvador, Ribeira Beach. The COI and 16S sequences were deposited in GenBank under accession numbers, KM192180–KM192188 and KM192197–KM192205, respectively. All COI sequences are identical. The 16S sequences showed one variable site, and the genetic distance between sequences varied from 0% to 0.2% (*p*-distance and K2P). See Molecular identity in *T. caribous* and *T. ceciliae* for an inter-specific comparison between *T. punctata* complex × *T. caribous* and *T. punctata* complex × *T. ceciliae*, respectively.

Distribution

Timarete punctata is a complex of species and it has been recorded throughout the Atlantic (western and eastern), Indian (western) and Pacific (western) oceans, e.g. [18,24,25,26]. Preliminary molecular analysis has shown that the Caribbean and South Atlantic material are composed of two distinct species and additional research is planned to elucidate the identity of both species.

Acknowledgments

This study would not have been possible without the collecting efforts of various colleagues listed in the material examined section. Drs B. Neuhaus (MfN), Geoff Keel and K. Osborn (USNM) kindly loaned several type and voucher material of south Atlantic cirratulids. Tina M. Carvalho (BEMF,

University of Hawaii) provided guidance with SEM procedure. Drs F. Barros and R. Johnsson (UFBA) provided laboratory and equipment support for sorting and photographing live specimens in Salvador, Brazil. Dr. Antônio M. Solé-Cava provided the facilities of Laboratório de Biodiversidade Molecular (UFRJ) for sequencing samples. Dr. J. Bailey-Brock (University of Hawaii) provided guidance and sugestions to a previous version of this paper. Dr. J. Blake and an anonymous reviewer carefully read a previous version and made considerable suggestions to improve this paper.

Author Contributions

Conceived and designed the experiments: WM VS PP. Performed the experiments: WM VS PP RE. Analyzed the data: WM VS. Contributed reagents/materials/analysis tools: WM VS PP. Contributed to the writing of the manuscript: WM VS PP RE.

References

1. Hartman O (1959) Catalogue of the Polychaetous Annelids of the World. Occas Pap Allan Hancock Foundation 23: 628 p.
2. Grube AE (1873) Die Familie der Cirratuliden. Jahres-Bericht der Schlesiche Gesellschaft fuer vaterlandische Cultur 50: 59–66.
3. Hansen GA (1882) Recherches sur les annélides recueillies par M. le professeur Édouard van Benedon pendant son voyage au Brésil et à la Plata. Memoires Couronnes et Memoires des Savants Etrangers publies par L'Academie Royale des Sciences, des Lettres et des Beaux-Arts de Belgique 44(3): 1–29.
4. Quatrefages A (1866) Histoire naturelle des Annelés marins et d'eau douce. Annélides et Géphyriens 2: 1–336.
5. Kinberg JGH (1866) Annulata nova. Öfversigt af Königlich Vetenskapsakademiens förhandlingar 23(9): 337–357.
6. Ehlers E (1897) Polychaeten. Hamburger Magalhaensische Sammelreise 1897: 1–148.
7. Treadwell AL (1932) Annelidos polychetos novos da Ilha de Sao Sebastiao. Rev Mus Paul 17(2): 1–20.
8. Amaral ACZ, Nallin SAH, Steiner TM (2006) Catálogo das espécies dos Annelida Polychaeta do Brasil. Available: http://www2.ib.unicamp.br/projbiota/bentos_marinho/catalogo_polychaeta2006.pdf. Accessed 22 July 2014.
9. Floyd R, Abebe E, Papert A, Blaxter M (2002) Molecular barcodes for soil nematode identification. Mol Ecol 11: 839–850. Doi:10.1046/j.1365-294X.2002.01485.x.
10. Folmer O, Black M, Hoeh W, Lutz R, Vrijenhoek R (1994) DNA primers for amplification of mitochondrial cytochrome c oxidase subunit I from diverse metazoan invertebrates. Mol Mar Biol Biotech 3(5): 294–299.
11. Palumbi SR, Martin AP, Romano S, McMillan WO, Stice L, et al. (1991) The simple fool's guide to PCR. Departament of Zoology Special Publication, University of Hawaii, Honolulu, HI.
12. Zanol J, Halanych KM, Struck TH, Fauchald K (2010) Phylogeny of the bristle worm family Eunicidae (Eunicida: Annelida) and the phylogenetic utility of noncongruent 16S, COI and 18S in combined analyses. Molec Phylogen Evol 55(2): 660–676.
13. Tamura K, Peterson D, Peterson N, Stecher G, Nei M, et al. (2011) MEGA5: Molecular Evolutionary Genetics Analysis using Maximum Likelihood, evolutionary distance, and Maximum Parsimony methods. Molec Biol Evol 28: 2731–2739.
14. Blake JA (1975) The larval development of Polychaeta from the northern California coast. I. *Cirriformia spirabrancha* (Family Cirratulidae). T Am Microsc Soc 94: 179–188.
15. Wilson DP (1936) The development of *Audouinia tentaculata* (Montagu). J Mar Biol Assoc UK 20: 567–579.
16. Grube AE (1859) Annulata Örstediana. Viden Medd Dansk Naturhist Köbenhavn: 105–120.
17. Kirkegaard JB (1981) Polychaetes from Saint Paul's Rocks, central Atlantic. Steenstrupia 7(11): 261–266.
18. Petersen ME (1999) Reproduction and development in Cirratulidae (Annelida: Polychaeta). In: A.W.C. Dorresteijn and W. Westheide (eds.). Reproductive strategies and developmental patterns in Annelids, p. 107–128. Hydrobiologia, Amsterdam.
19. Çinar ME (2009) Alien polychaete species (Annelida: Polychaeta) on the southern coast of Turkey (Levantine Sea, eastern Mediterranean), with 13 new records for the Mediterranean Sea. J Nat Hist 43 (37–38): 2283–2328.
20. Hartman O (1942) The identity of some marine annelid worms in the United States National Museum. Proc U S Natl Mus 92(3142): 101–140.
21. Hartman O (1948) The marine annelids erected by Kinberg. With some notes on some other types in the Swedish State Museum. Arkiv för Zoologi 42A(1): 1–137.
22. Grube AE (1869) Beschreibungen neuer oder weniger bekannter von Hrn. Ehrenberg gesammelter Anneliden des rothen Meeres. Monat Kon Preus Akad Wiss Berlin: 484–521.
23. Hartman O (1956) Polychaetous annelids erected by Treadwell, 1891 to 1948, together with a brief chronology. Bull Am Mus Nat Hist 109: 239–310.
24. Çinar ME (2007) Re-description of *Timarete punctata* (Polychaeta: Cirratulidae) and its occurrence in the Mediterranean Sea. Sci Mar 71: 755–764.
25. Day JH (1962) Polychaeta from several localities in the western Indian Ocean. Proc Zool Soc Lond 139: 627–656.
26. Hartman O (1966) Polychaetous annelids of the Hawaiian Islands. Occas Pap Bern P Bishop Mus 23: 163–252.
27. Verrill AE (1900) Additions to the Turbellaria, Nemertina, and Annelida of the Bermudas, with revisions of some New England genera and species. Trans Conn Acad Arts Sci 10(2): 595–671.
28. Magalhães WF, Bailey-Brock JH (2010) Redescription of *Cirriformia crassicollis* (Kinberg, 1866) and *Timarete hawaiensis* (Hartman, 1956) new combination, (Polychaeta: Cirratulidae), endemic polychaetes to the Hawaiian Islands. Zootaxa 2625: 53–62.
29. Blake JA (1996) 8. Family Cirratulidae Ryckholdt, 1851. Including a revision of the genera and species from the eastern North Pacific. In: J. A. Blake, B. Hilbig and P.H. Scott (eds.), Taxonomic Atlas of the Benthic Fauna of the Santa Maria Basin and the Western Santa Barbara Channel. Volume 6– The Annelida Part 3, Polychaeta: Orbiniidae to Cossuridae. Santa Barbara Museum of Natural History, 263–384. Santa Barbara, California.
30. Imajima M & Hartman O (1964) The polychaetous annelids of Japan. Occas Pap Allan Hancock Found, 26(1–2): 1–452.
31. Montagu G (1808) Description of several marine animals found on the south coast of Devonshire. Trans Linn Soc Lond 9: 81–114.
32. Treadwell AL (1936) Polychaetous annelids from the vicinity of Nonsuch Island, Bermuda. Zoologica 21(1): 49–68.
